# Identification of Microalgae, Evaluation of Water Purification Efficiency, and Optimization of Large-Scale Production Conditions Based on Recirculating Aquaculture Systems

**DOI:** 10.3390/plants15192950

**Published:** 2026-09-27

**Authors:** Zekui Ou, Gan Yang, Jiancha Luo, Dan Huang, Quan Gan, Shiyu Chen, Siyi Lin

**Affiliations:** 1 College of Life Science, Hengyang Normal University, Hengyang 421000, China; 2 Hunan Key Laboratory for Conservation and Utilization of Biological Resources in the Nanyue Mountainous Region, Hengyang 421000, China

**Keywords:** microalgae biotechnology, algae biomass production, optimization of abiotic conditions, water purification application, land-based recirculating aquaculture systems

## Abstract

Water purification is the most crucial process for recirculating aquaculture systems (RASs), while microalgae exhibit significant potential for nutrient removal from aquaculture wastewater. Four microalgal species with removal capacities for ammonia nitrogen (NH_4_^+^-N), nitrite nitrogen (NO_2_-N) and total phosphorus (TP) were isolated and identified from the land-based RASs, namely, *Kirchneriella cornuta* HY-1, *Desmodesmus abundans* HY-1, *Coccomyxa* sp. HY-1 and *Oocystis* sp. HY-1. The growth kinetics were characterized through measurements of cell density, chlorophyll content, and dry biomass in the in situ wastewater. Based on zeta potential, the flocculation efficiencies of seven agents were evaluated. Preservation parameters were optimized under nutrient-supplemented conditions with protective agents. Under large-scale culture conditions, over a 7-day period, the highest NH_4_^+^-N removal efficiency of 87.20 ± 0.56% was achieved by *C*. sp. HY-1, the highest NO_2_-N removal efficiency (85.63 ± 2.56%) was obtained by *O*. sp. HY-1, and the maximum TP removal efficiency (71.19 ± 3.35%) was exhibited by *K*. *cornuta* HY-1. Moreover, the harvest period could be reached after about 14 days of cultivation. FeCl_3_ was identified as the optimal harvesting flocculant for all four species. Optimal preservation conditions were determined as 25 °C and 31.25 μmol·m^−2^·s^−1^ for *D*. *abundans* HY-1, *C.* sp. HY-1, and *O.* sp. HY-1, and 25 °C and 18.75 μmol·m^−2^·s^−1^ for *K. cornuta* HY-1. This work provides theoretical guidance for large-scale microalgal production and confirms the technical feasibility of microalgae-driven in situ wastewater remediation.

## 1. Introduction

Aquaculture contributes significantly to global food security and economic development. However, traditional open-net cage farming can negatively impact the environment through eutrophication, and the stocks are vulnerable to diseases, harmful algal blooms, and parasites from surrounding waters [1,2]. As an alternative, land-based recirculating aquaculture systems (RASs) are fish farming facilities where fish are raised in water tanks under controlled conditions. Wastewater is treated through various filtration and purification mechanisms to remove metabolic waste from the cultured fish before being recycled [3]. RASs enable substantial reductions in water consumption and improvements in waste management and nutrient recycling, thereby rendering intensive fish production compatible with environmental sustainability [4]. Despite these advantages, intensive land-based RASs generate substantial volumes of nitrogen- and phosphorus-rich wastewater [5,6]. This wastewater can cause severe environmental issues such as nutrient enrichment, oxygen depletion, and loss of biodiversity, thereby constraining the sustainability and efficiency of high-density land-based aquaculture [7,8]. Accordingly, integration of microalgal biotechnology into RASs represents a promising approach to addressing this challenge.

Previous studies have confirmed that microalgae can effectively mitigate wastewater pollution by assimilating nitrogen and phosphorus through photosynthesis, thereby achieving in situ water purification [9]. Integration of microalgal biotechnology into land-based RASs can significantly reduce freshwater consumption, substantially decrease nutrient emissions, and enhance system carrying capacity and aquaculture density by increasing dissolved oxygen and reducing ammonia and nitrite concentrations. These benefits collectively improve the operational stability, environmental sustainability, and economic efficiency of land-based RASs [10,11,12]. Green algae, as photoautotrophic eukaryotes, convert solar radiation into biomass containing high-value compounds using widely available natural resources, including carbon dioxide, water, and inorganic salts [13,14,15]. Moreover, they are characterized by high photosynthetic efficiency, rapid reproduction rates, and strong environmental adaptability [16]. These photosynthetic microorganisms serve as promising renewable energy sources with nearly zero net carbon dioxide emissions, as carbon dioxide released during their use is equivalent to the amount fixed from the atmosphere by microalgae [17]. Additionally, they play crucial roles in ecological water purification and maintain the balance of aquatic ecosystems. For instance, *Scenedesmus* sp. exhibits antioxidant, immune-enhancing, detoxification-promoting, and digestive-improving functions [18,19]. *Oocystis* sp. can improve water quality, maintain ecological balance, efficiently utilize nitrogen and phosphorus nutrients, and release oxygen [20]. *Kirchneriella* sp. and *Chlorella vulgaris* demonstrate strong capacity for water remediation, exhibiting particularly efficient uptake and removal of nitrogen and phosphorus, thereby effectively mitigating eutrophication [21,22].

Despite the recognized potential, systematic comparisons of cultivation, harvesting, and preservation techniques for microalgae locally isolated from RAS wastewater remain limited. In recent years, researchers have conducted extensive studies focusing on three key technologies: efficient cultivation, low-cost harvesting, and low-loss preservation [23,24,25]. However, most studies have been conducted on model or commercially available strains rather than microalgae locally isolated from RAS wastewater, leaving a critical gap in strain-specific technical optimization. To address this gap, this study selected the isolated microalgal strains described above as study organisms. The present study focused on large-scale cultivation, comparison of harvesting methods, and optimization of preservation techniques, aiming to screen for the optimal harvesting and preservation schemes for different microalgae suitable for wastewater purification scenarios in land-based RASs.

## 2. Results

### 2.1. Phenotypic Characteristics and Identification of Microalgal Taxa

**Figure 1:**
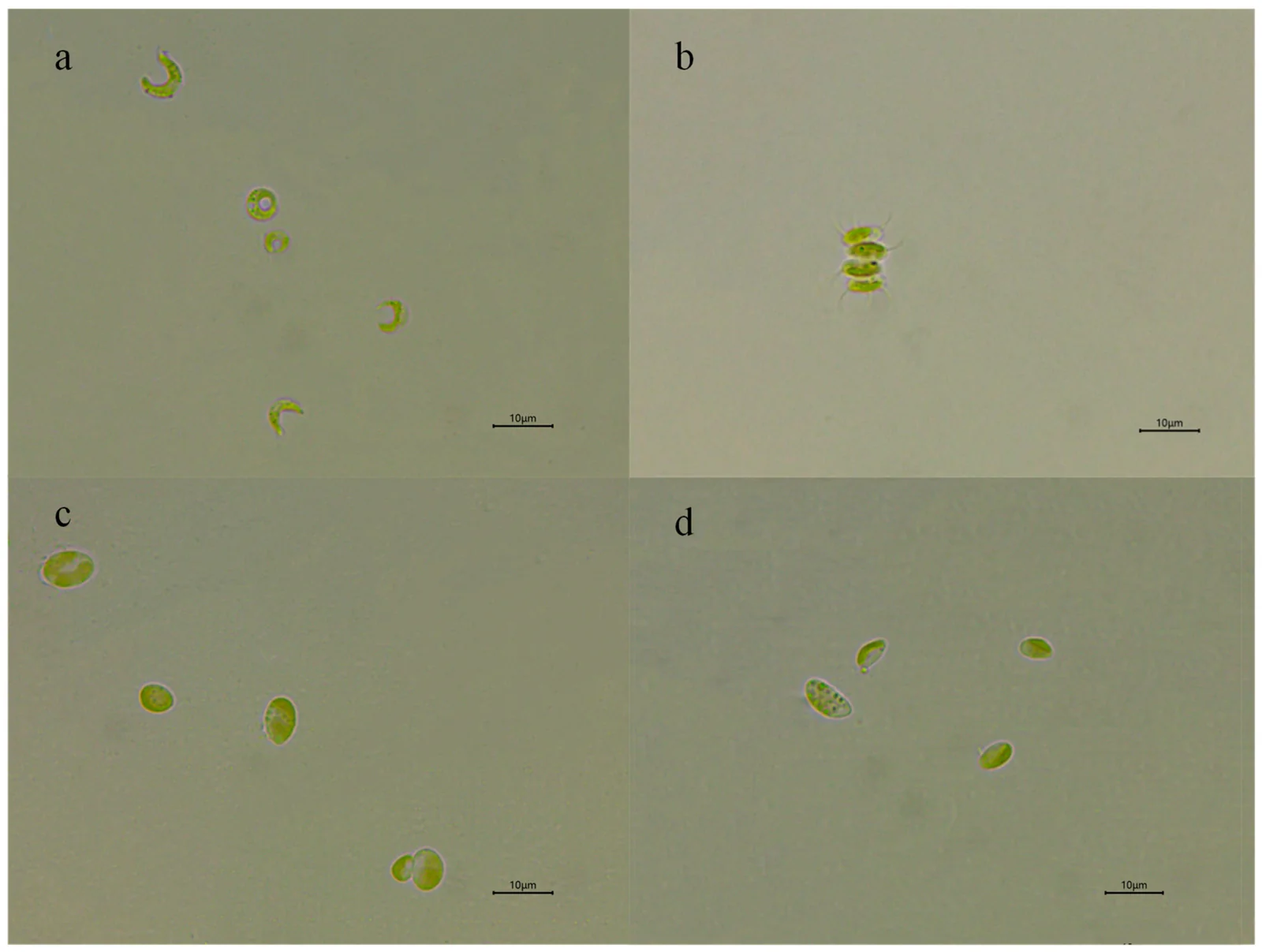
Morphological characteristics of four microalgae. (**a**) *K. cornuta* HY-1; (**b**) *D. abundans* HY-1; (**c**) *C.* sp. HY-1; (**d**) *O.* sp. HY-1.

All identified microalgae belonged to Chlorophyta. Among these, both strains HYNU202601 and HYNU202602 were members of Sphaeropleales (Table 1). Strain HYNU202601 formed colonies of four or eight cells, most of which were embedded in a mucilaginous envelope and were planktonic; cells were crescent-shaped or hoof-shaped, with pointed ends, and the chloroplasts were radially distributed around the cell, filling the entire cell except for the middle part of the concave side of the cell. Cells were 7–20 μm in length and 3–8 μm in width (Figure 1a). Strain HYNU202601 morphological descriptions were consistent with the characteristics of *Kirchneriella* algae. Strain HYNU202602 formed coenobia of four cells arranged in a single linear series. Cells were arranged in a row in a straight line; cells were oval to elongated oval, with blunt rounded ends. Each cell of the group had a long spine at both the upper and lower ends, and the middle part of the outer wall usually had two to three spines. The two middle cells had no spines or had very short spiny protrusions at the upper and lower ends. The width of the group of four cells was 14 to 24 μm; individual cells measured 7 to 13 μm in length and 3.5 to 4 μm in width (Figure 1b). The strain HYNU202602 morphological descriptions were in line with the characteristics of *Desmodesmus* algae.

The strains HYNU202601 and HYNU202602 showed high ITS sequence similarity with the type species *Kirchneriella cornuta* and *Desmodesmus abundans,* respectively. They were members of the family Sphaeropleales. More specifically, strain HYNU202601 (640 bp ITS 1, 5.8S ribosomal RNA, and ITS 2 sequences gene fragment) showed 99.29% sequence identity at 88% query coverage with the type strain *Kirchneriella cornuta* CFR 8-01/FW and was identified as *Kirchneriella cornuta* HY-1, while strain HYNU202602 (582 bp fragment) showed 100% sequence similarity at 100% query coverage with the type strain *Desmodesmus abundans* TAU-MAC 3110 and was identified as *Desmodesmus abundans* and named as *Desmodesmus abundans* HY-1.

Both the strains HYNU202603 and HYNU202604 were members of Trebouxiophyceae. The strain HYNU202603 formed large, irregular mucilaginous colonies in which cells were randomly dispersed; the group cells were oval in shape, straight or slightly curved, with chromatophores surrounding them and in a long sheet-like form, located on one side of the cell. Cells were 7 to 20 μm in length and 3.5 to 8 μm in width (Figure 1c). The above morphological descriptions were in line with the characteristics of *Coccomyxa* algae. Further, the strain (621 bp fragment) showed 99.52% sequence similarity at 99% query coverage with the type strain *Coccomyxa* sp. LXD and was classified as *Coccomyxa*. Finally, based on the morphology and gene sequence identification of the strain, the strain HYNU202603 was identified and named as *Coccomyxa* sp. HY-1.

The strain HYNU202604 was oval-shaped, composed of two, four, or eight cells encapsulated within a partially gelatinized and enlarged mother cell wall, or it could be a single cell, floating; cells were oval or slightly ovoid, with broad rounded ends, the pigment granules were sheet-like, and usually there was only one pigment granule at the early stage, while after maturation there were two to four pigment granules, each with a pyrenoid. Cells were 10 to 30 μm in length and 9 to 15 μm in width (Figure 1d). The strain (410 bp fragment) showed 100% sequence similarity at 100% query coverage with the type strain *Oocystis* sp. CAUP H1110 and was classified as *Oocystis*. Finally, based on the morphology and gene sequence identification of the strain, the strain HYNU202604 was identified and named as *Oocystis* sp. HY-1.

### 2.2. Water Purification Effect of Microalgae

#### 2.2.1. Removal Efficiency of Microalgae on NH_4_^+^-N

As shown in Table 2, initial concentration of NH_4_^+^-N in the recirculating aquaculture system (RAS) was 0.919 ± 0.008 mg·L^−1^. NH_4_^+^-N concentration of the wastewater in the control group only decreased from 0.917 ± 0.004 mg·L^−1^ to 0.864 ± 0.002 mg·L^−1^. The removal efficiency was 5.78 ± 0.61%, significantly lower than experimental groups (*p* < 0.05). Over the cultivation period, all four microalgae consistently reduced NH_4_^+^-N concentrations. By D5, *C*. sp. HY-1 achieved the lowest residual NH_4_^+^-N (0.293 ± 0.007 mg·L^−1^), followed by *K. cornuta* HY-1 (0.422 ± 0.024 mg·L^−1^). *D. abundans* HY-1 (0.595 ± 0.005 mg·L^−1^) and *O.* sp. HY-1 (0.581 ± 0.005 mg·L^−1^) did not differ significantly (*p* > 0.05) and were both inferior to *K. cornuta* HY-1 on D5 (*p* < 0.05). From D6 to D7, uptake rates of the four microalgae tended to stabilize. Although *C.* sp. HY-1 and *D. abundans* HY-1 showed comparable concentrations through D5, *C.* sp. HY-1 exhibited a markedly higher removal efficiency from D5 to D7. NH_4_^+^-N concentration in the *C.* sp. HY-1 group decreased to 0.120 ± 0.007 mg·L^−1^, achieving a removal efficiency of 87.20 ± 0.56%, significantly higher than those of *D. abundans* HY-1 (56.60 ± 0.44%), *O.* sp. HY-1 (49.33 ± 3.19%), and *K. cornuta* HY-1 (65.30 ± 2.46%) (*p* < 0.05). These results demonstrate a distinct advantage of *C.* sp. HY-1 in NH_4_^+^-N purification.

#### 2.2.2. Removal Efficiency of Microalgae on NO_2_-N

As shown in Table 3, under conditions where NO_2_-N concentration in recirculating aquaculture wastewater was 7.820 ± 0.107 mg·L^−1^, four microalgae effectively reduced NO_2_-N levels, with concentrations decreasing consistently over cultivation time. The NO_2_-N in the control group decreased from 7.843 ± 0.011 mg·L^−1^ to 7.294 ± 0.017 mg·L^−1^, and the removal efficiency was 7.00 ± 0.33%, which was significantly lower than the experimental groups (*p* < 0.05). Among the four types of microalgae, *D. abundans* HY-1 exhibited the highest NO_2_-N removal efficiency from D2 to D4, with its NO_2_-N concentration significantly lower than those of the other groups (*p* < 0.05). From D5 to D6, NO_2_-N removal performance of *D. abundans* HY-1 and *O.* sp. HY-1 groups was comparable. However, by D7, NO_2_-N concentration in the *O.* sp. HY-1 group had decreased to 1.146 ± 0.223 mg·L^−1^, while that in the *D. abundans* HY-1 group had declined more slowly to 1.695 ± 0.057 mg·L^−1^. *K. cornuta* HY-1 showed relatively poor performance in removing NO_2_-N, with its concentration on D7 reaching 3.564 ± 0.097 mg·L^−1^, significantly higher than those in the other groups (*p* < 0.05). On D7, NO_2_-N concentration in the *C.* sp. HY-1 group was 2.414 ± 0.122 mg·L^−1^. After 7 days, the removal efficiency for NO_2_-N by *O.* sp. HY-1 was 85.63 ± 2.56%, significantly higher than those of *D. abundans* HY-1 (78.28 ± 0.49%), *K. cornuta* HY-1 (53.36 ± 1.04%), and *C.* sp. HY-1 (69.20 ± 1.42%) (*p* < 0.05). Thus, *O.* sp. HY-1 exhibited a distinct advantage in NO_2_-N removal.

#### 2.2.3. Removal Efficiency of Microalgae on TP

As shown in Table 4, under an initial TP concentration of 1.502 ± 0.020 mg·L^−1^ in the RAS wastewater, all four microalgae effectively reduced TP levels, with concentrations decreasing steadily over the cultivation period. No significant difference in initial TP concentration was observed among groups (*p* > 0.05). The TP in the wastewater of the control group decreased from 1.505 ± 0.001 mg·L^−1^ to 1.419 ± 0.002 mg·L^−1^, and the removal efficiency was 5.71 ± 0.05%, which was significantly lower than the experimental groups (*p* < 0.05). From D1 to D4, *D. abundans* HY-1 and *C.* sp. HY-1 showed similar phosphorus removal efficiencies (*p* > 0.05), whereas the TP concentrations in the *K. cornuta* HY-1 and *O.* sp. HY-1 groups were significantly lower than those in the *D. abundans* HY-1 and *C.* sp. HY-1 groups (*p* < 0.05). On D5, TP concentration in *C.* sp. HY-1 group was significantly higher than the other three groups (*p* < 0.05), while the concentrations in *D. abundans* HY-1, *K. cornuta* HY-1, and *O.* sp. HY-1 groups were similar (*p* > 0.05). From D5 to D7, the *K. cornuta* HY-1 group maintained the highest removal efficiency for TP compared to the other three groups. By the end of cultivation (D7), TP concentration in *K. cornuta* HY-1 group dropped to 0.430 ± 0.051 mg·L^−1^, achieving a removal efficiency of 71.19 ± 3.35%, significantly higher than those of *O.* sp. HY-1 (60.43 ± 3.20%), *D. abundans* HY-1 (56.07 ± 1.13%), and *C.* sp. HY-1 (51.36 ± 0.24%) (*p* < 0.05). Thus, *K. cornuta* HY-1 exhibited a distinct advantage in TP purification.

### 2.3. Microalgae Growth Characteristics in Large-Scale Cultivation

#### 2.3.1. Cell Density and Cell Dry Weight Measurement

**Figure 2:**
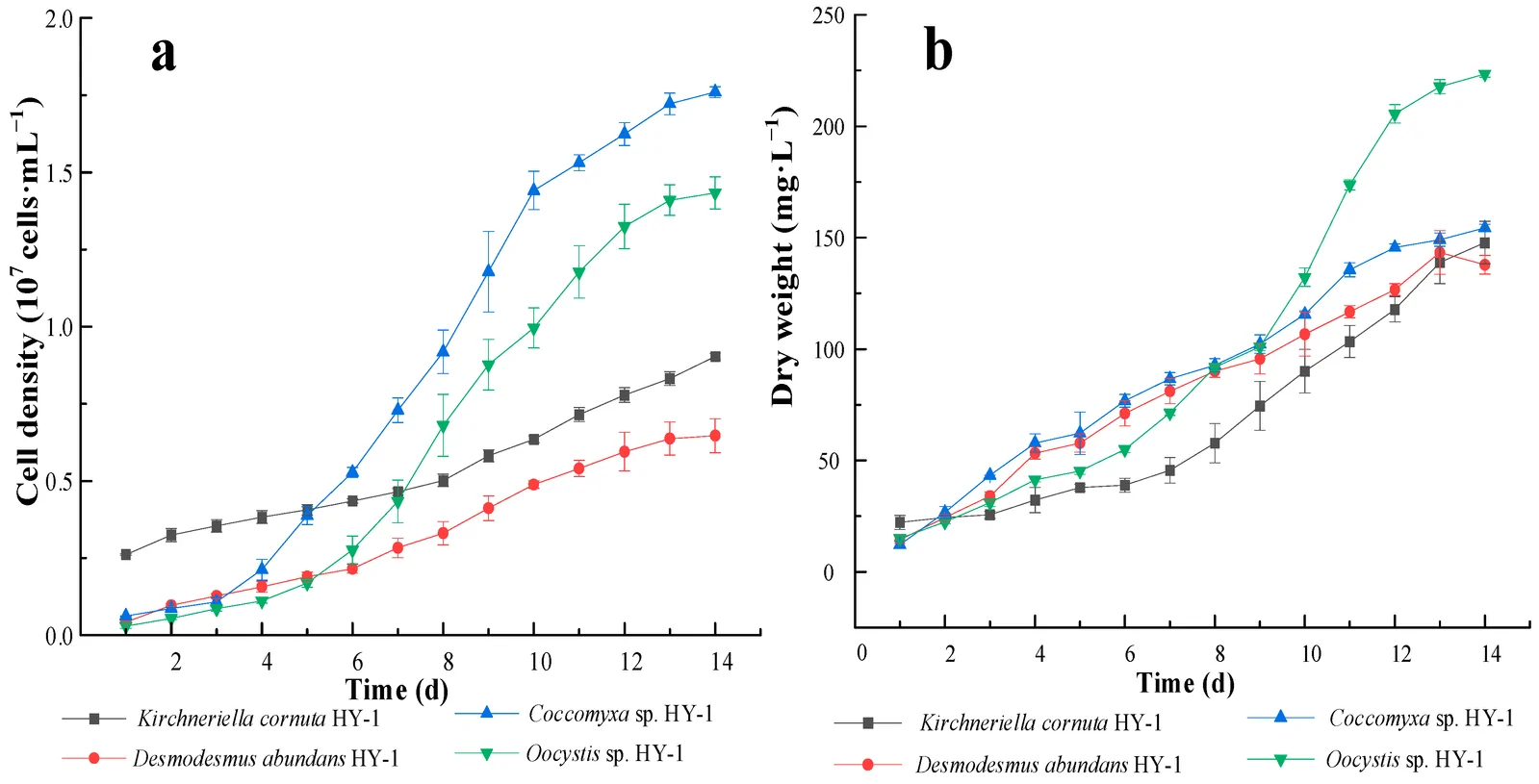
Algal density and dry cell weight variation curves of the four microalgae. (**a**) Cell densities of four microalgae; (**b**) Dry weight of four microalgae.

As shown in Figure 2a, the cell density of the four microalgae strains exhibited an increasing trend throughout the experimental period, but there were differences in the growth rates of the four strains. During the initial stage, the growth of each algal group was relatively slow, indicating that the algal species were in an environmental adaptation phase, adjusting their physiological states to the new environment. After a period of time, the growth pattern of the algal species changed to exponential growth. The exponential growth curve equation of *C*. sp. HY-1 during D2–D9 was Y = e^(−2.09987+0.2791x)^ (*R^2^* = 0.97974), and μ = 0.2791 d^−1^; for *K. cornuta* HY-1 during D3–D14, the exponential growth curve equation was Y = e^(−3.47796+0.09029x)^ (*R*^2^ = 0.9936), μ = 0.09029 d^−1^; for *O*. sp. HY-1 during D3–D14, the exponential growth curve equation was Y = e^(0.14048+0.23334x)^ (*R*^2^ = 0.947), μ = 0.23334 d^−1^; and during D4–D12, for *D. abundans* HY-1, Y = e^(−2.18152+0.17849x)^ (*R*^2^ = 0.99157), μ = 0.17849 d^−1^. After 14 days of cultivation, the growth of microalgae tended to slow down. The cell density of *C.* sp. HY-1 was 1.760 ± 0.018 × 10^7^ cells·mL^−1^, which was significantly higher than that of *O.* sp. HY-1 (1.433 ± 0.052 × 10^7^ cells·mL^−1^), *K. cornuta* HY-1 (1.005 ± 0.039 × 10^7^ cells·mL^−1^), and *D. abundans* HY-1 (0.647 ± 0.054 × 10^7^ cells·mL^−1^) (*p* < 0.05).

As shown in Figure 2b, the dry weights of the four microalgae exhibited exponential growth, indicating a high degree of consistency between the dry weight growth trend and the exponential equation. The exponential growth curve equation of *C*. sp. HY-1 during D1–D10 was Y = e^(3.33118+0.14577x)^ (*R*^2^ = 0.94241), μ = 0.14577 d^−1^. For *K. cornuta* HY-1 during D4–D14, the exponential growth curve equation was Y = e^(3.32925+0.0771x)^ (*R*^2^ = 0.98391), μ = 0.0771 d^−1^. For *O*. sp. HY-1 during D5–D12, the exponential growth curve equation was Y = e^(3.592+0.21816x)^ (*R*^2^ = 0.99428), μ = 0.21816 d^−1^. During D4–D13, for *D. abundans* HY-1, it was Y = e^(3.93685+0.10296x)^ (*R*^2^ = 0.98951), μ = 0.10296 d^−1^. During the initial stage of the experiment (D1–D7), growth patterns of *C.* sp. HY-1 and *D. abundans* HY-1 were similar, with dry weights of cells significantly higher than those of *O.* sp. HY-1 and *K. cornuta* HY-1 (*p* < 0.05). From D5 to D12, *O.* sp. HY-1 began to grow exponentially, consistent with the cell density trend observed in Figure 2a; by D14, the dry weight of *O.* sp. HY-1 cells reached 223.55 ± 1.47 mg·L^−1^, which was significantly higher than those of *C.* sp. HY-1 (154.44 ± 1.57 mg·L^−1^), *K. cornuta* HY-1 (147.78 ± 9.56 mg·L^−1^), and *D. abundans* HY-1 (143.33 ± 4.15 mg·L^−1^), demonstrating a strong advantage in biomass accumulation (*p* < 0.05). Notably, the higher dry weight of *O*. sp. HY-1, despite its lower cell density relative to *C*. sp. HY-1, was attributed to its larger cell volume. Overall, *K. cornuta* HY-1, *D. abundans* HY-1, and *C.* sp. HY-1 maintained relatively consistent growth patterns throughout the experimental period, while *O.* sp. HY-1 exhibited a distinct late-stage growth advantage.

#### 2.3.2. Photosynthetic Pigment Content Measurement

As shown in Table 5, Table 6 and Table 7, the photosynthetic pigment content of the four microalgae all exhibited an increasing trend. Fitting the data for chlorophyll a, chlorophyll b, and carotenoid content yielded coefficients of determination (*R*^2^) all above 0.96 (Table 8). The growth rates of chlorophyll a and chlorophyll b contents of *D. abundans* HY-1 and *K. cornuta* HY-1 accelerated over time. According to the fitting curve equation, at D14, the growth rates of chlorophyll a and chlorophyll b reached 0.067 and 0.038 μg·mL^−1^·d^−1^ for *D. abundans* HY-1, and 0.047 and 0.052 μg·mL^−1^·d^−1^ for *K. cornuta* HY-1, respectively. These rates were higher than the growth rate of carotenoid content (0.011 μg·mL^−1^·d^−1^ and 0.016 μg·mL^−1^·d^−1^). The growth rate of photosynthetic pigment content in *C*. sp. HY-1 and *O*. sp. HY-1 slowed down over time. The growth rates of chlorophyll a, chlorophyll b, and carotenoid content reached 0.079, 0.007, and 0.039 μg·mL^−1^·d^−1^ for *C*. sp. HY-1, and 0.036, 0.022, and 0.008 μg·mL^−1^·d^−1^ for *O*. sp. HY-1, respectively. These indicated that *D*. *abundans* HY-1 and *K*. *cornuta* HY-1 maintained active photosynthetic pigment synthesis throughout the cultivation period, whereas *C*. sp. HY-1 and *O*. sp. HY-1 exhibited decelerating pigment accumulation toward the later stage, possibly reflecting a transition toward stationary phase or a shift toward non-pigment biomass accumulation. From the perspective of the composition of photosynthetic pigments, after 14 days, the chlorophyll a content of *C.* sp. HY-1 reached 1.482 ± 0.020 μg·mL^−1^, chlorophyll b (1.082 ± 0.222 μg·mL^−1^), and carotenoid (0.578 ± 0.072 μg·mL^−1^), all significantly higher than those of the other three strains (*p* < 0.05), consistent with the highest cell density observed for *C.* sp. HY-1; the chlorophyll a content of *K. cornuta* HY-1 reached 0.422 ± 0.027 μg·mL^−1^, chlorophyll b was 0.415 ± 0.077 μg·mL^−1^, and carotenoid was 0.159 ± 0.016 μg·mL^−1^; the chlorophyll a content of *D. abundans* HY-1 reached 0.598 ± 0.086 μg·mL^−1^, chlorophyll b was 0.607 ± 0.059 μg·mL^−1^, and carotenoid was 0.184 ± 0.007 μg·mL^−1^; and the chlorophyll a content of *O.* sp. HY-1 reached 0.547 ± 0.012 μg·mL^−1^, chlorophyll b was 0.485 ± 0.027 μg·mL^−1^, and carotenoid was 0.133 ± 0.023 μg·mL^−1^. Thus, *C.* sp. HY-1 maintained a high level of activity and rapid growth and reproduction characteristics under this cultivation condition. At D14, the chlorophyll content of *C*. sp. HY-1 was significantly higher than that of the other three microalgae. However, according to the fitting curve equation, photosynthetic pigment contents of *D. abundans* HY-1 and *K. cornuta* HY-1 might exceed that of *C*. sp. HY-1 with the time increase.

### 2.4. Microalgae Harvesting

#### 2.4.1. Flocculation Effect of Chitosan on Microalgae

**Figure 3:**
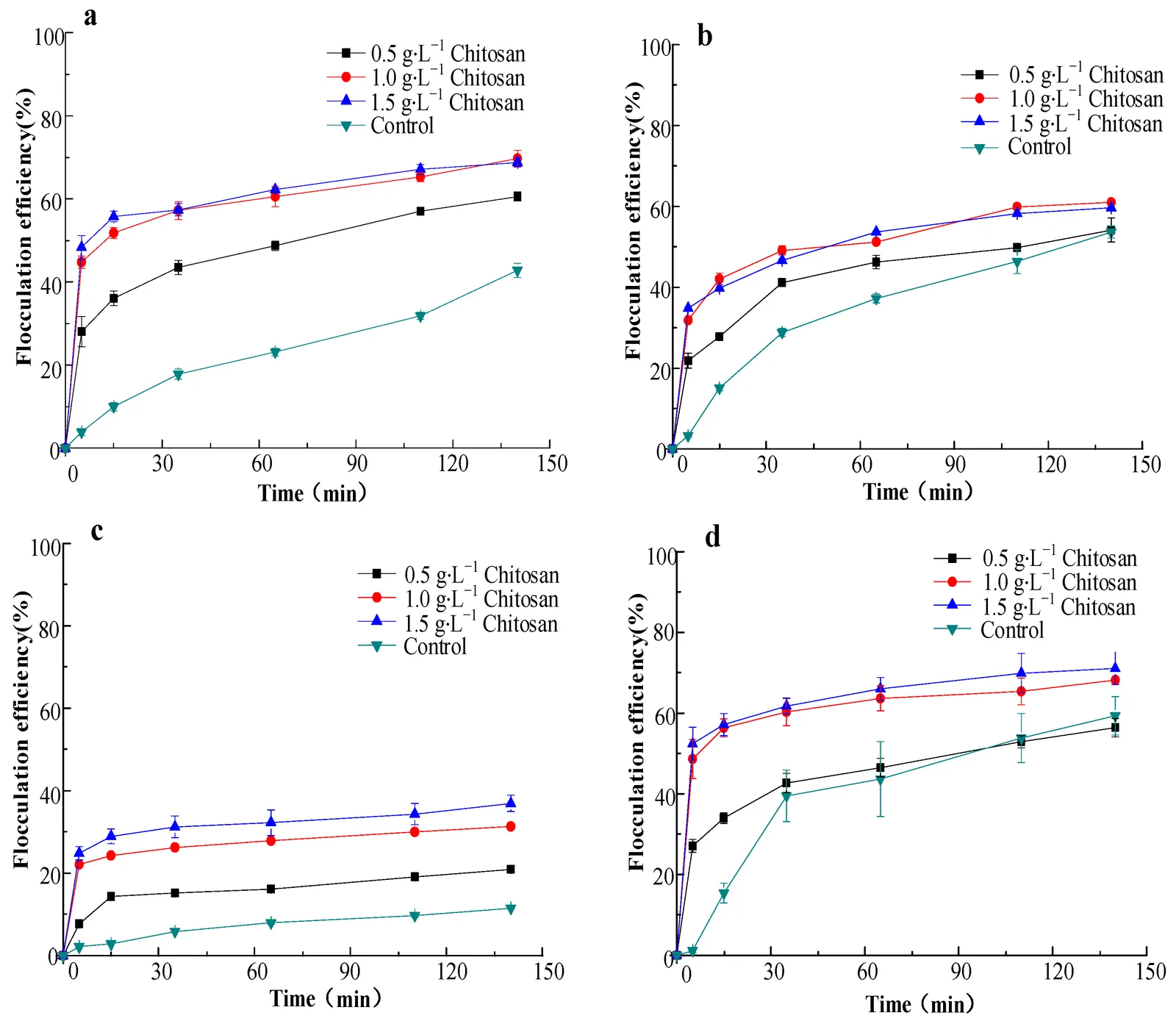
Flocculation efficiency of chitosan for the four microalgae: (**a**) *O*. sp. HY-1; (**b**) *C*. sp. HY-1; (**c**) *K. cornuta* HY-1; (**d**) *D. abundans* HY-1.

The flocculation efficiency of chitosan increased with concentration for all four microalgae (Figure 3). From Figure 3a,b, after 145 min, 1.0 g·L^−1^ and 1.5 g·L^−1^ chitosan achieved flocculation efficiency of over 60% for both *O.* sp. HY-1 and *C.* sp. HY-1. At 1.0 g·L^−1^ and 1.5 g·L^−1^ chitosan, zeta potentials were 5.07 ± 0.06 and 5.23 ± 0.13 mV for *C*. sp. HY-1, and 4.47 ± 0.13 and 4.50 ± 0.15 mV for *O.* sp. HY-1, respectively, which showed significant differences from the control group (*p* < 0.05). However, the flocculation-enhancing effect of chitosan on *C.* sp. HY-1 was limited, as gravity sedimentation alone already achieved 55.61 ± 1.50%. Flocculation efficiency of chitosan on *K. cornuta* HY-1 was significantly lower than that of the other three algae (*p* < 0.05), with a maximum of only 34.12 ± 1.95% at 1.5 g·L^−1^ (Figure 3c); zeta potentials of 0.5, 1.0 and 1.5 g·L^−1^ chitosan groups were −6.28 ± 0.18 mV, 5.93 ± 0.08 and 5.68 ± 0.16 mV (as absolute values). As shown in Figure 3d, at 145 min, 1.5 g·L^−1^ chitosan had a significantly higher flocculation efficiency for *D. abundans* HY-1 compared to the other three strains, reaching 71.02 ± 0.04%, with a corresponding zeta potential of 4.21 ± 0.12 mV. From Table 9, the pH decreased with the increase in chitosan concentration. Chitosan had a certain flocculation efficiency on all four strains, albeit with varying efficacy. Among the four microalgae, the highest efficiency was observed for *D. abundans* HY-1 (*p* < 0.05).

#### 2.4.2. Flocculation Effect of Alkaline Agents on Microalgae

**Figure 4:**
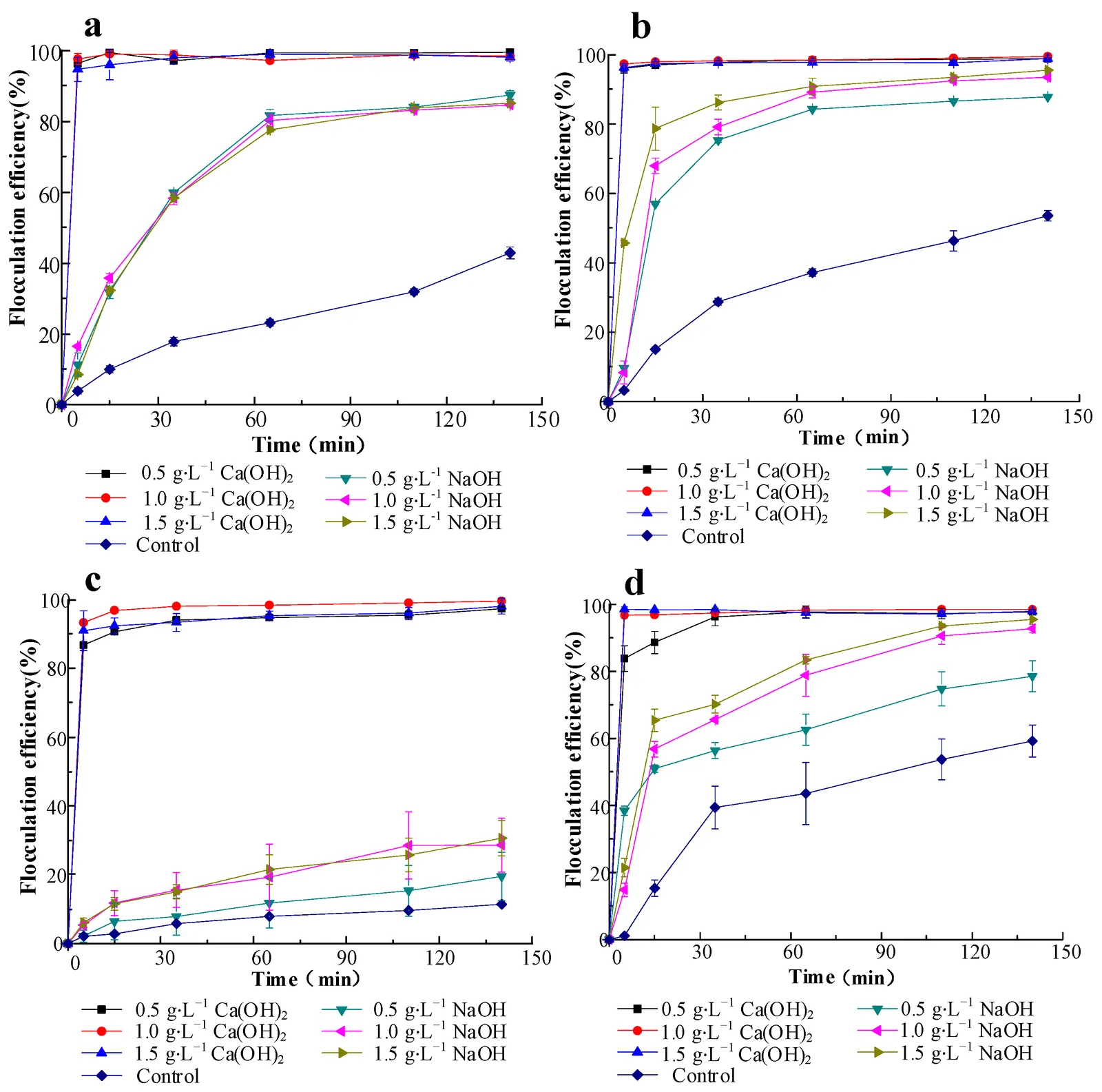
Flocculation efficiency of alkaline flocculant for the four microalgae: (**a**) *O*. sp. HY-1; (**b**) *C*. sp. HY-1; (**c**) *K. cornuta* HY-1; (**d**) *D. abundans* HY-1.

As demonstrated in Figure 4a,c, all concentration groups of Ca(OH)_2_ for *K. cornuta* HY-1 and *O.* sp. HY-1 exhibited high flocculation efficiency at 145 min, with no significant differences among the three concentrations (*p* > 0.05). From Table 10, after the addition of Ca(OH)_2_, zeta potentials of the *K. cornuta* HY-1 and *O.* sp. HY-1 groups were, respectively, from −1.46 to 0.83 mV and from −0.97 to −1.08 mV; the potentials were relatively close to zero. Thus, all Ca(OH)_2_ concentration groups showed high flocculation efficiency. For NaOH, the efficiency among different concentration groups for *K. cornuta* HY-1 was similar, while for *O.* sp. HY-1, the 0.5 g·L^−1^ group had lower efficiency, and the other two groups had higher efficiency.

As demonstrated in Figure 4b,d, for *D. abundans* HY-1 and *C.* sp. HY-1, the 1.0 g·L^−1^ group efficiency was not significantly different from the 1.5 g·L^−1^ group efficiency in the Ca(OH)_2_ groups (*p* > 0.05). In Table 10, zeta potentials of the 1.0 g·L^−1^ and 1.5 g·L^−1^ groups were −0.95 ± 0.22 and 1.03 ± 0.21 mV for *D. abundans* HY-1, and −0.59 ± 0.18 and 0.72 ± 0.18 mV for *C.* sp. HY-1 (*p* > 0.05). For *D. abundans* HY-1 in the NaOH group, at 35 min, the 1.0 g·L^−1^ group efficiency was not significantly different from the 1.5 g·L^−1^ group efficiency (*p* > 0.05). For *C.* sp. HY-1 in the NaOH group, at 5 min, the 0.5 g·L^−1^ group efficiency was not significantly different from the 1.0 g·L^−1^ group efficiency (*p* > 0.05), while the 1.0 g·L^−1^ group efficiency was significantly different from the 1.5 g·L^−1^ group efficiency (*p* < 0.05), with the 1.5 g·L^−1^ group having the highest efficiency (*p* < 0.05). After 5 min, the efficiency gradually increased and stabilized at 145 min, showing significant differences compared to the other two groups (*p* < 0.05). The flocculation efficiencies of 1.5 g·L^−1^ NaOH groups of *D. abundans* HY-1 and *C.* sp. HY-1 were 95.57 ± 0.14 and 95.07 ± 0.40%, with corresponding zeta potentials (absolute values) of 33.31 ± 0.21 and 33.38 ± 0.18 mV. From Table 10, the pH increases with the increase in Ca(OH)_2_ and NaOH concentration.

Overall, for all four strains, flocculation efficiencies of the 1.0 and 1.5 g·L^−1^ Ca(OH)_2_ groups both exceeded 95%, with no significant difference between them (*p* > 0.05), and were significantly higher than that of the corresponding NaOH groups (*p* < 0.05). Therefore, the optimal flocculation concentration range for all four microalgal strains was 1.0–1.5 g·L^−1^ Ca(OH)_2_.

#### 2.4.3. Flocculation Effect of Aluminum Salts on Microalgae

**Figure 5:**
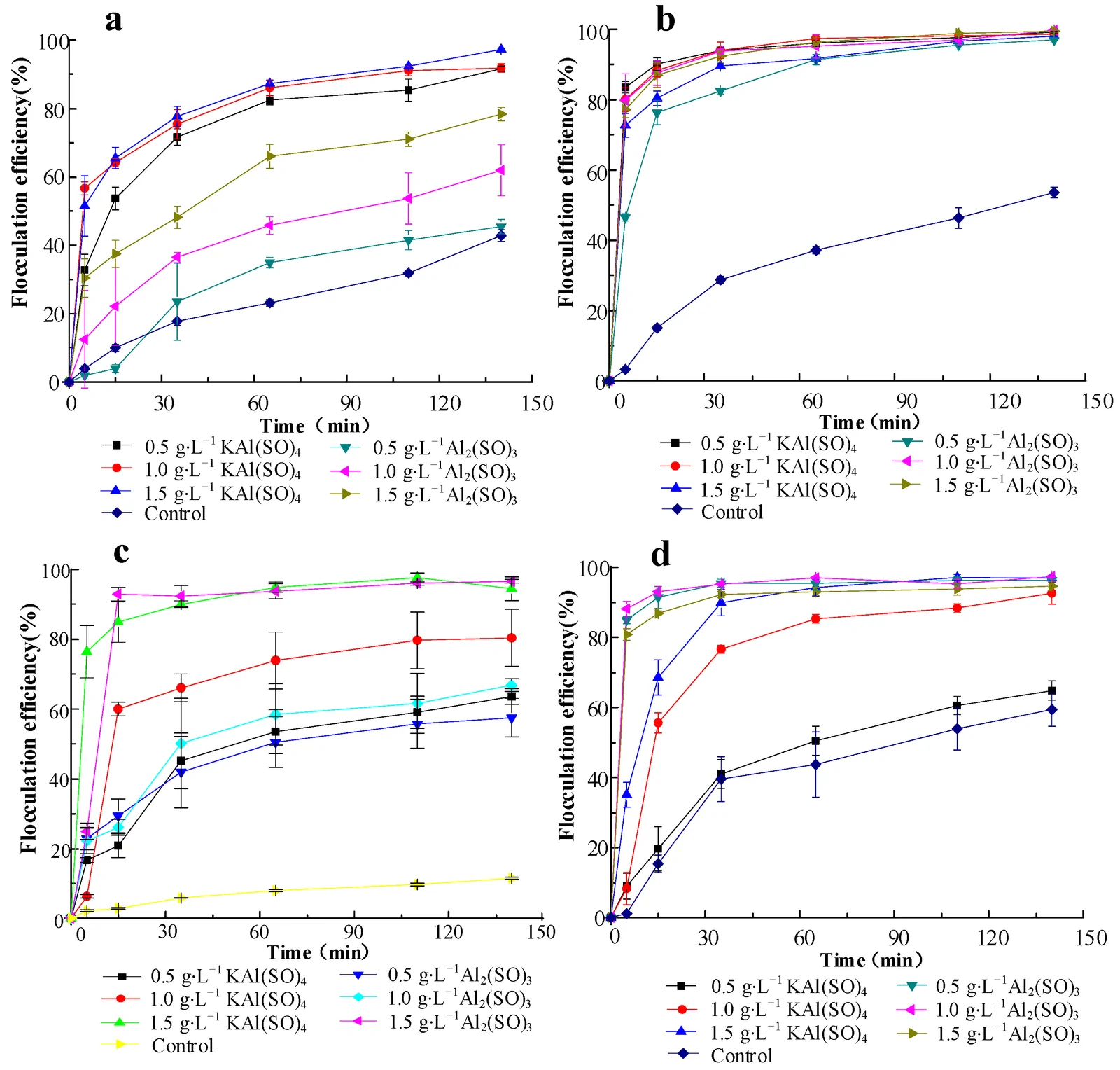
Flocculation efficiency of aluminum salts for the four microalgae: (**a**) *O*. sp. HY-1; (**b**) *C*. sp. HY-1; (**c**) *K. cornuta* HY-1; (**d**) *D. abundans* HY-1.

As demonstrated in Figure 5a,b, the flocculation efficiency of both the *O.* sp. HY-1 and *C.* sp. HY-1 showed an upward trend in the KAl(SO_4_)_2_ group and Al_2_(SO_4_)_3_ group. In Figure 5a, KAl(SO_4_)_2_ group efficiency was significantly higher than Al_2_(SO_4_)_3_ group efficiency, reaching over 91.59% (*p* < 0.05). In the KAl(SO_4_)_2_ group, at 145 min, the 1.5 g·L^−1^ group’s efficiency was significantly different from the 0.5 g·L^−1^ and 1.0 g·L^−1^ groups’ efficiency, reaching 97.26 ± 0.22% (*p* < 0.05). Zeta potentials of 0.5, 1.0 and 1.5 g·L^−1^ were −1.92 ± 0.18, −1.86 ± 0.18, and 1.19 ± 0.14 mV. In Figure 5b, neither flocculation efficiency nor zeta potential differed significantly between the KAl(SO_4_)_2_ and Al_2_(SO_4_)_3_ groups (*p* > 0.05), reaching 98.72 ± 0.95%.

As demonstrated in Figure 5c,d, the flocculation efficiency of both *K. cornuta* HY-1 and *D. abundans* HY-1 in the KAl(SO_4_)_2_ and the Al_2_(SO_4_)_3_ groups showed an upward trend. In Figure 5c, among the KAl(SO_4_)_2_ and Al_2_(SO_4_)_3_ groups, the 1.5 g·L^−1^ group was significantly higher than the 1.0 g·L^−1^ and 0.5 g·L^−1^ groups (*p* < 0.05), reaching 96.43 ± 0.52%. Zeta potential of the 1.5 g·L^−1^ group was lower than the 1.0 g·L^−1^ and 0.5 g·L^−1^ groups (*p* < 0.05). No significant difference in flocculation efficiency was detected between 1.5 g·L^−1^ KAl(SO_4_)_2_ and Al_2_(SO_4_)_3_ groups (*p* > 0.05), and commonality existed in their zeta potentials (*p* > 0.05). In Figure 5d, the 1.5 g·L^−1^ group under the KAl(SO_4_)_2_ treatment had significantly higher flocculation efficiency than the other groups of 1.0 g·L^−1^ and 0.5 g·L^−1^ (*p* < 0.05), reaching 96.67 ± 0.71%. Zeta potentials of the 1.0 g·L^−1^ and 0.5 g·L^−1^ groups were lower than the 1.5 g·L^−1^ group (*p* < 0.05), whereas there were no significant differences among the three groups in the Al_2_(SO_4_)_3_ group (*p* > 0.05). From Table 11, the pH decreased with the increase in aluminum salts concentration.

#### 2.4.4. Flocculation Effects of Iron Salts on Microalgae

**Figure 6:**
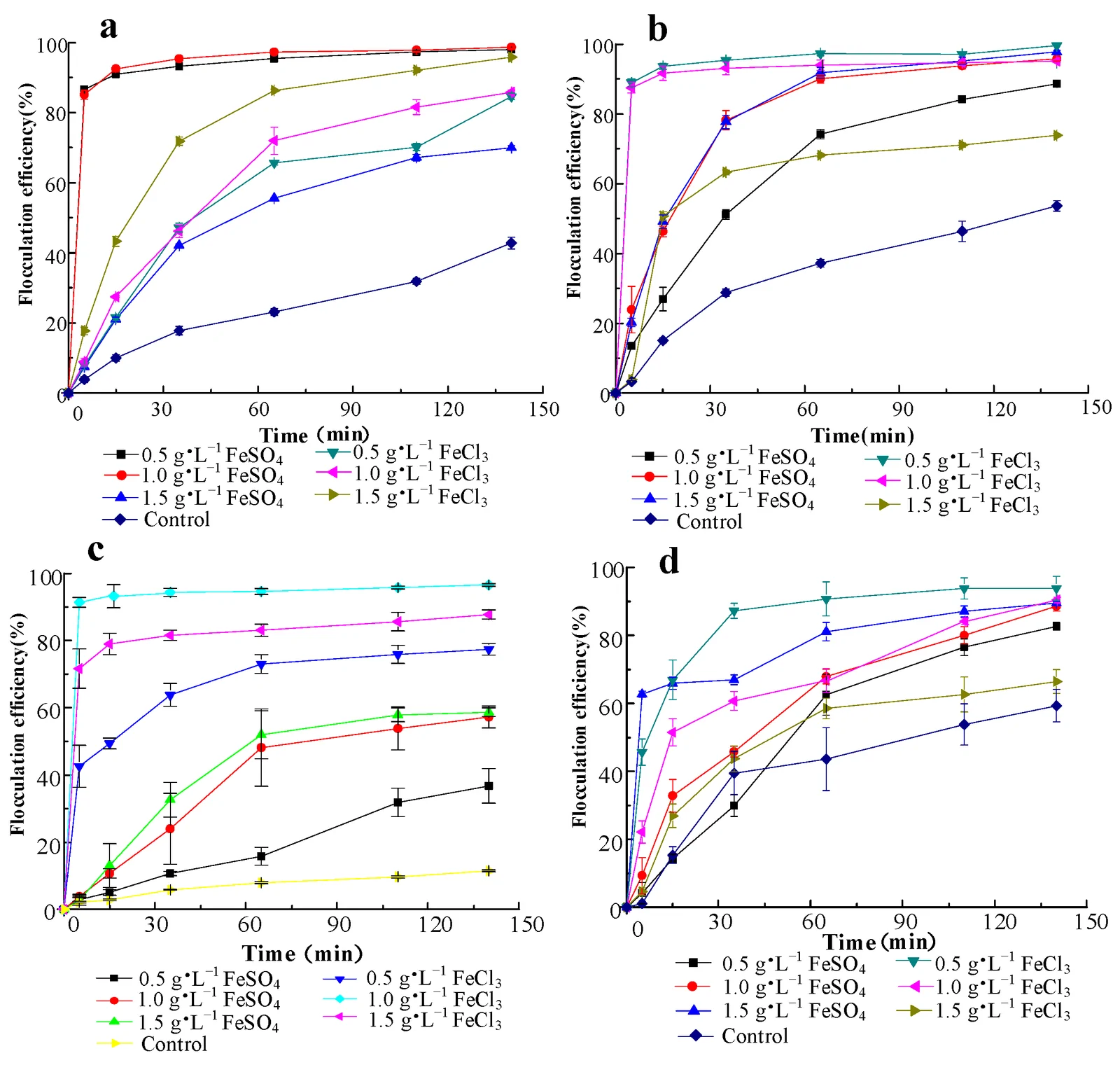
Flocculation efficiency of iron salts for the four microalgae: (**a**) *O*. sp. HY-1; (**b**) *C*. sp. HY-1; (**c**) *K. cornuta* HY-1; (**d**) *D. abundans* HY-1.

As shown in Figure 6a,c, flocculation efficiencies of *O.* sp. HY-1 and *K. cornuta* HY-1 increased over time in both the FeSO_4_ and FeCl_3_ groups at each concentration. In Figure 6a, no significant differences in efficiency or zeta potential were observed between the 0.5 and 1.0 g·L^−1^ FeSO_4_ groups (*p* > 0.05). On the other hand, both had flocculation efficiency (98.35 ± 0.50%) significantly higher than the 1.5 g·L^−1^ group (*p* < 0.05), and the zeta potentials for both (−2.5 ± 0.25 mV) were significantly lower than the 1.5 g·L^−1^ group (*p* < 0.05). In Figure 6c, the flocculation efficiency of FeCl_3_ was significantly higher than that of FeSO_4_ groups (*p* < 0.05), and zeta potentials of FeCl_3_ were significantly lower than FeSO_4_ groups (*p* < 0.05). Flocculation efficiency at 1.0 g·L^−1^ FeCl_3_ was significantly higher than those at 0.5 and 1.5 g·L^−1^ FeCl_3_, reaching 96.50 ± 0.41% (*p* < 0.05). Zeta potentials of *K. cornuta* HY-1 at 0.5, 1.0 and 1.5 g·L^−1^ FeCl_3_ were −3.25 ± 0.22 mV, 1.27 ± 0.17 mV and 2.09 ± 0.16 mV (*p* < 0.05). Therefore, concentrations of 1.0 to 1.5 g·L^−1^ FeCl_3_ could be selected.

As demonstrated in Figure 6b,d, flocculation efficiency of *C.* sp. HY-1 and *D. abundans* HY-1 in both the FeSO_4_ and FeCl_3_ groups increased over time. In Figure 6b, during the period from 0 to 65 min, the flocculation efficiency in the FeCl_3_ group at 0.5 and 1.0 g·L^−1^ was significantly higher than for the 1.5 g·L^−1^ group (*p* < 0.05). The zeta potential of 0.5 g·L^−1^ FeCl_3_ was −0.61 ± 0.15 mV, which was significantly lower than that in other subgroups. The flocculation efficiency in the FeSO_4_ group reached its maximum at 1.0 and 1.5 g·L^−1^ at 145 min. Therefore, concentrations of 0.5 to 1.0 g·L^−1^ FeCl_3_ were selected. In Figure 6d, at 145 min, flocculation efficiency in the FeCl_3_ group at 0.5 and 1.0 g·L^−1^ was significantly higher than that at 1.5 g·L^−1^ FeCl_3_ (*p* < 0.05), reaching 93.04 ± 2.68%. The zeta potential of 0.5 g·L^−1^ FeCl_3_ was −1.48 ± 0.17 mV, which was significantly lower than that in other subgroups. Flocculation efficiency in the FeSO_4_ group at 1.0 and 1.5 g·L^−1^ was significantly higher than for 0.5 g·L^−1^ (*p* < 0.05), reaching 89.02 ± 1.15%. There was no significant difference between 1.0 g·L^−1^ and 1.5 g·L^−1^ FeSO_4_ (*p* > 0.05). From Table 12, the pH decreases with the increase in iron salts concentration.

### 2.5. Microalgae Preservation

**Figure 7:**
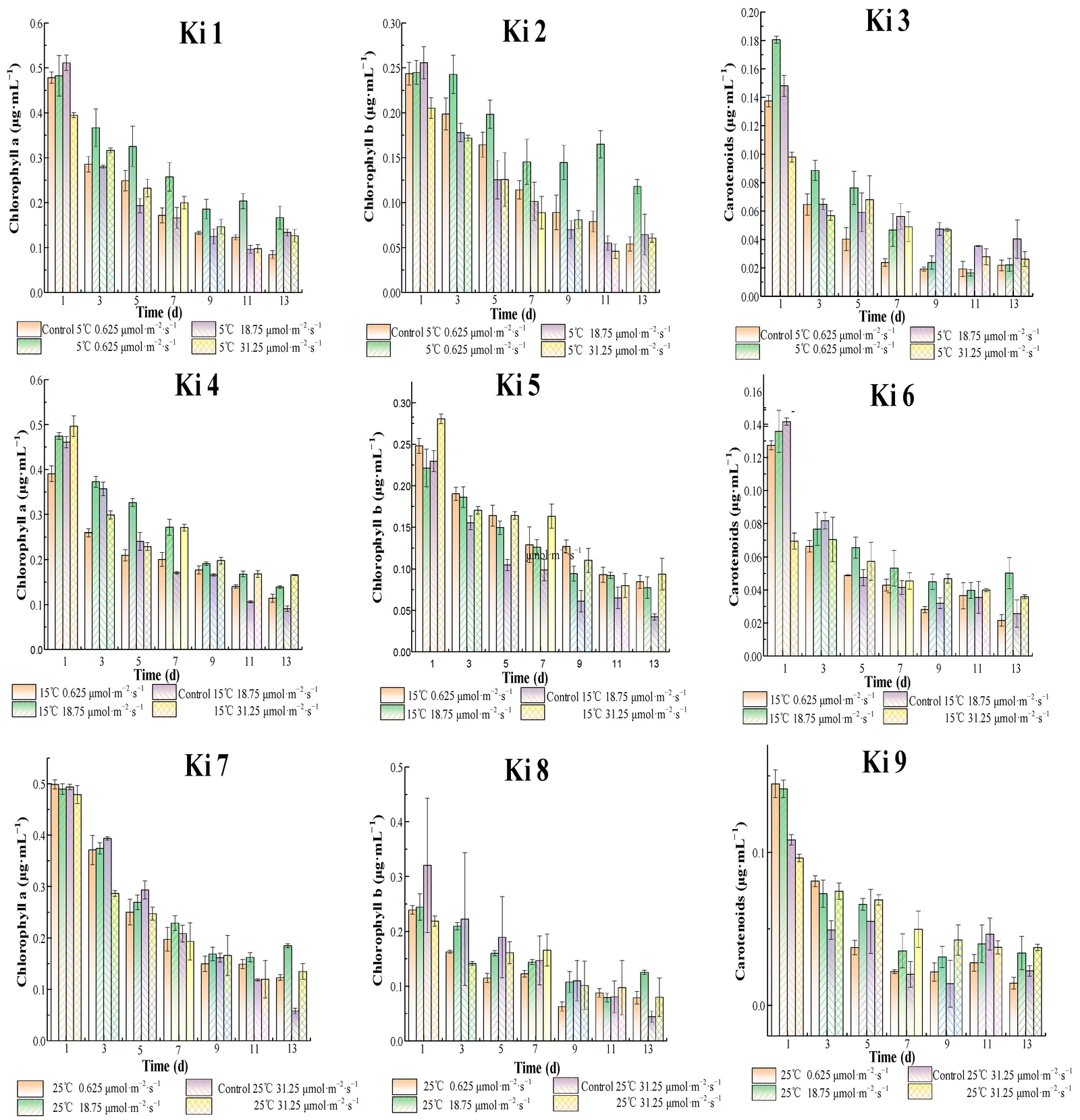
Changes in photosynthetic pigment content of *K. cornuta* HY-1 (Ki) under different preservation conditions. (Ki 1–9: Changes in photosynthetic pigment content of *K. cornuta* HY-1 at 5, 15, and 25 °C).

**Figure 8:**
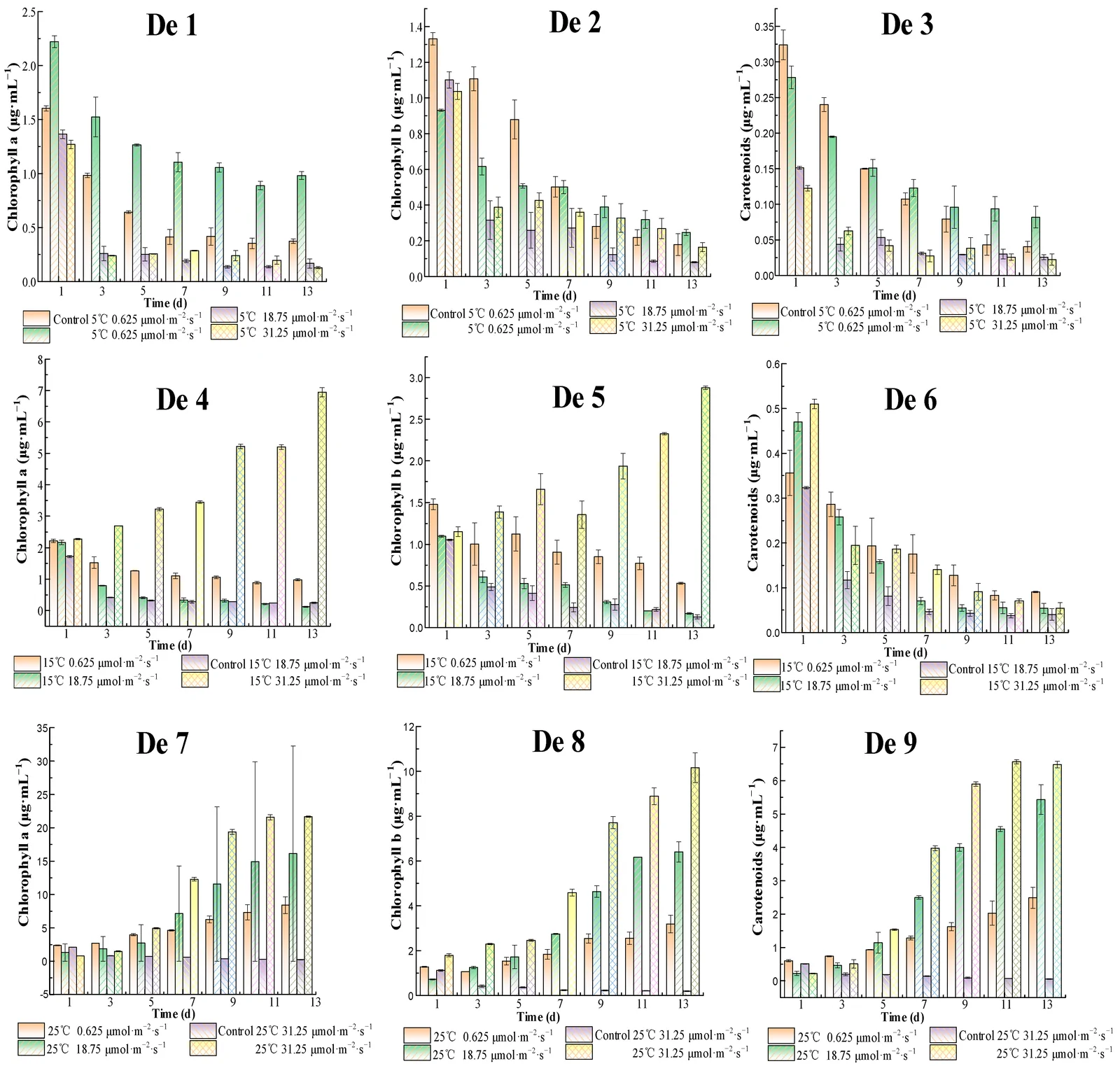
Changes in photosynthetic pigment content of *D. abundans* HY-1 (De) under different preservation conditions. (De 1–9): Changes in photosynthetic pigment content of *D. abundans* HY-1 at 5, 15, and 25 °C.

**Figure 9:**
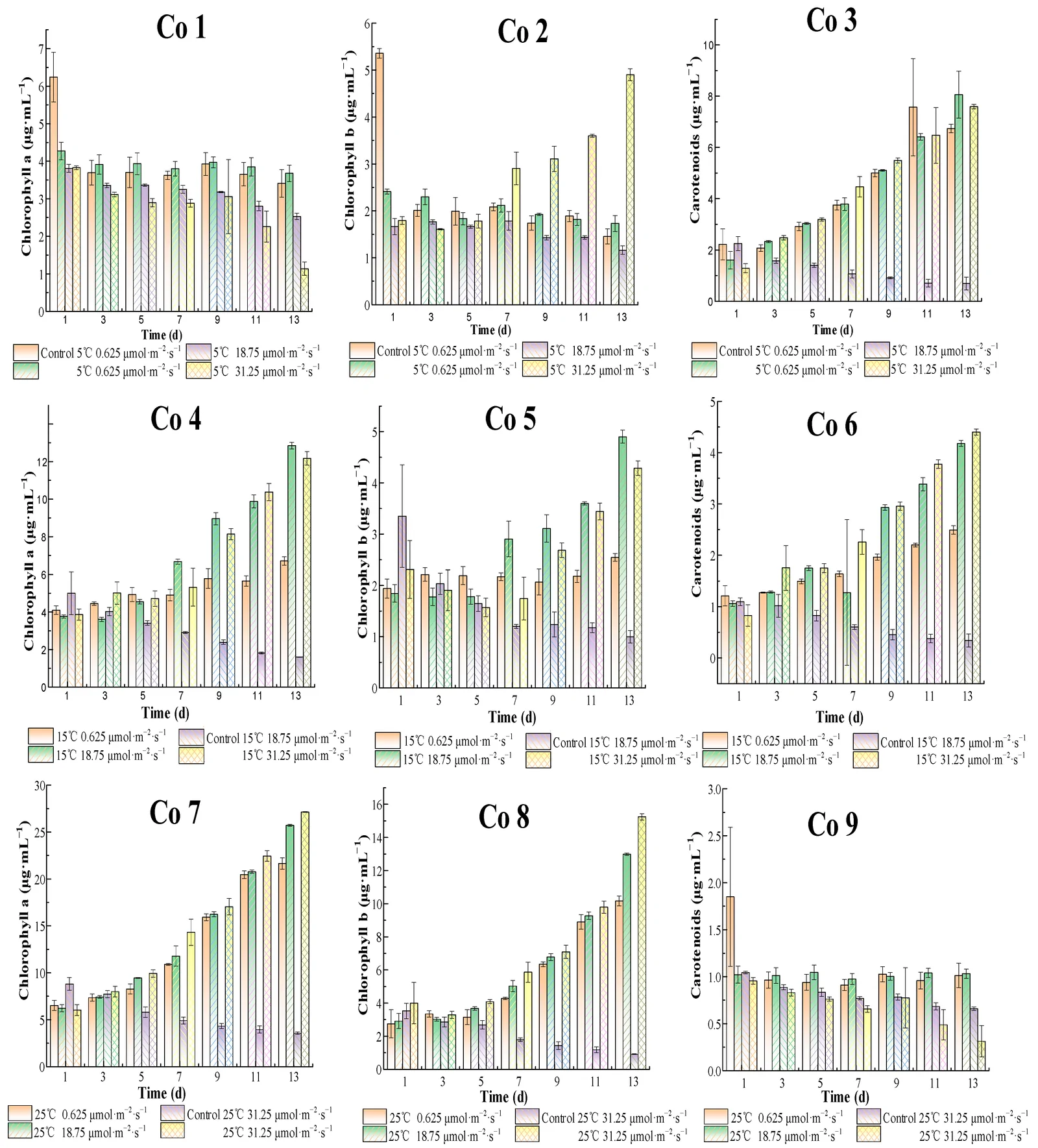
Changes in photosynthetic pigment content of *C*. sp. HY-1 (Co) under different preservation conditions. (Co 1–9): Changes in photosynthetic pigment content of *C*. sp. HY-1 at 5, 15, and 25 °C.

**Figure 10:**
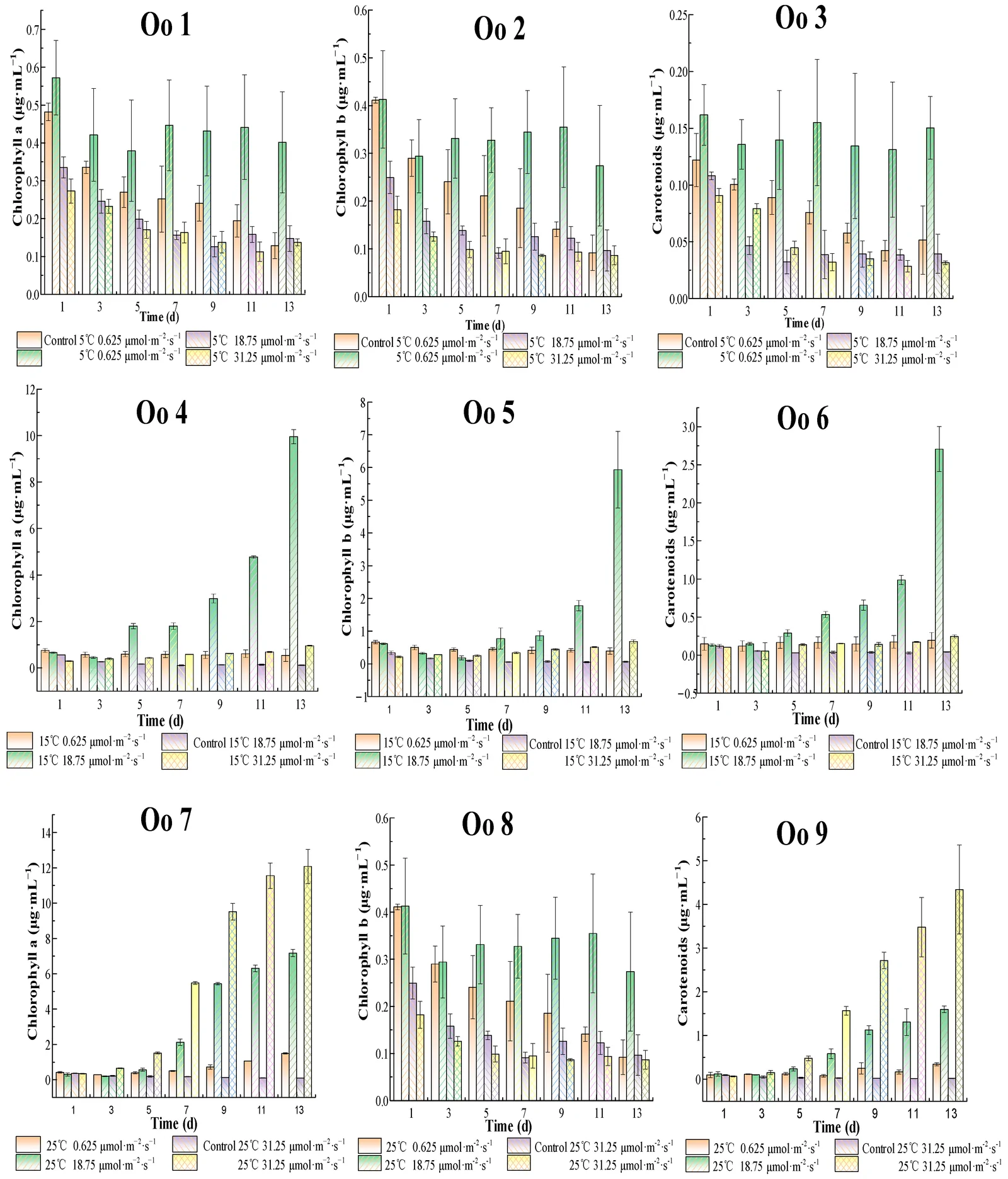
Changes in photosynthetic pigment content of *O*. sp. HY-1 (Oo) under different preservation conditions. (Oo 1–9): Changes in photosynthetic pigment content of *O*. sp. HY-1 at 5, 15, and 25 °C.

The preservation conditions (temperature, light) influenced the photosynthetic pigment dynamics of the four microalgae over the 14-day period in the protective-agent groups (Figure 7, Figure 8, Figure 9 and Figure 10). For *K. cornuta* HY-1, two-way ANOVA revealed a significant interaction effect of light intensity and temperature on chlorophyll a content (F = 11.697, *p* < 0.05). From Figure 7, simple effects analysis showed that, at 25 °C, the chlorophyll a content in the 18.75 μmol·m^−2^·s^−1^ group was 0.19 ± 0.05 μg·mL^−1^, which was significantly higher than those in the 0.625 and 31.25 μmol·m^−2^·s^−1^ groups (*p* < 0.05). At 15 °C, however, the 31.25 μmol·m^−2^·s^−1^ group had the highest chlorophyll a content (0.14 ± 0.001 μg·mL^−1^), significantly exceeding the 0.625 and 18.75 μmol·m^−2^·s^−1^ groups (*p* < 0.05). At 5 °C, no significant difference was detected among light intensity groups, with chlorophyll a averaging 0.15 ± 0.02 μg·mL^−1^ (*p* > 0.05). Further simple effects analysis at the optimal light level (18.75 μmol·m^−2^·s^−1^) showed that chlorophyll a content at 25 °C was significantly higher than at 15 °C and 5 °C (*p* < 0.05). A significant interaction effect of light intensity and temperature was also observed for chlorophyll b (F = 5.646, *p* < 0.05). Simple effects analysis identified the optimal preservation conditions as 25 °C + 18.75 μmol·m^−2^·s^−1^, under which chlorophyll b content reached 0.13 ± 0.003 μg·mL^−1^. In contrast, no significant interaction effect was detected for carotenoids (F = 1.482, *p* = 0.249), indicating that the combined effects of light intensity and temperature on carotenoid content were not statistically significant. A significant main effect of light intensity on carotenoid content was detected (F = 14.546, *p* < 0.05), whereas the main effect of temperature (F = 1.762, *p* = 0.2) was not significant. Across all temperature levels, carotenoid content at 0.625 μmol·m^−2^·s^−1^ (0.20 ± 0.006 μg·mL^−1^) was significantly lower than at 31.25 μmol·m^−2^·s^−1^ (0.33 ± 0.006 μg·mL^−1^) and 18.75 μmol·m^−2^·s^−1^ (0.42 ± 0.13 μg·mL^−1^). No significant difference was detected between 31.25 and 18.75 μmol·m^−2^·s^−1^ (*p* > 0.05). Finally, compared with the initial values before preservation, the chlorophyll a, chlorophyll b, and carotenoid content was reduced by 67.76%, 66.43%, and 75.82%, respectively, under the best condition.

For *D. abundans* HY-1, two-way ANOVA revealed a significant interaction effect of light intensity and temperature on chlorophyll a content (F = 2107.90, *p* < 0.05). From Figure 8, simple effects analysis showed a crossover interaction pattern: at 5 °C, the 0.625 μmol·m^−2^·s^−1^ group had the highest chlorophyll a content (0.33 ± 0.04 μg·mL^−1^), significantly higher than the 18.75 and 31.25 μmol·m^−2^·s^−1^ groups (*p* < 0.05); at 15 °C and 25 °C, however, the 31.25 μmol·m^−2^·s^−1^ group yielded the highest chlorophyll a content, reaching 8.72 ± 0.05 μg·mL^−1^ at 15 °C and 26.13 ± 0.25 μg·mL^−1^ at 25 °C, both significantly exceeding the 0.625 and 18.75 μmol·m^−2^·s^−1^ groups (*p* < 0.05). Further simple effects analysis at the optimal light level (31.25 μmol·m^−2^·s^−1^) confirmed that chlorophyll a content at 25 °C was significantly higher than those at 15 °C and 5 °C (*p* < 0.05). Significant interaction effects were also observed for chlorophyll b (F = 246.75, *p* < 0.05) and carotenoids (F = 3390.61, *p* < 0.05). Simple effects analysis identified 25 °C + 31.25 μmol·m^−2^·s^−1^ as the optimal preservation conditions, under which chlorophyll b and carotenoid contents reached 12.52 ± 0.52 μg·mL^−1^ and 8.07 ± 0.65 μg·mL^−1^, respectively.

For *C*. sp. HY-1, two-way ANOVA revealed a significant interaction effect of light intensity and temperature on chlorophyll a content (F = 168.78, *p* < 0.05). From Figure 9, simple effects analysis showed that, at 5 °C, the 0.625 μmol·m^−2^·s^−1^ group had the highest chlorophyll a content (3.78 ± 0.22 μg·mL^−1^), significantly higher than the 18.75 and 31.25 μmol·m^−2^·s^−1^ groups (*p* < 0.05); at 15 °C, no significant difference was detected between the 18.75 and 31.25 μmol·m^−2^·s^−1^ groups (12.85 ± 0.18 and 12.17 ± 0.36 μg·mL^−1^, respectively), both of which were significantly higher than the 0.625 μmol·m^−2^·s^−1^ group (*p* < 0.05); at 25 °C, the 31.25 μmol·m^−2^·s^−1^ group yielded the highest chlorophyll a content (27.12 ± 0.04 μg·mL^−1^), significantly exceeding the 0.625 and 18.75 μmol·m^−2^·s^−1^ groups (*p* < 0.05). Further simple effects analysis at the optimal light level for chlorophyll a (31.25 μmol·m^−2^·s^−1^) confirmed that chlorophyll a content at 25 °C was significantly higher than those at 15 °C and 5 °C (*p* < 0.05). Significant interaction effects were also observed for chlorophyll b (F = 145.46, *p* < 0.05) and carotenoids (F = 10.13, *p* < 0.05). For chlorophyll b and carotenoids, simple effects analysis identified 25 °C and 31.25 μmol·m^−2^·s^−1^ as the optimal conditions, under which chlorophyll b and carotenoid contents reached 15.24 ± 0.19 μg·mL^−1^ and 7.59 ± 0.08 μg·mL^−1^, respectively.

For *O*. sp. HY-1, two-way ANOVA revealed a significant interaction effect of light intensity and temperature on chlorophyll a content (F = 310.33, *p* < 0.05). From Figure 10, simple effects analysis revealed a temperature-dependent light optima gradient: at 5 °C, the 0.625 μmol·m^−2^·s^−1^ group had the highest chlorophyll a content (0.40 ± 0.13 μg·mL^−1^), significantly higher than the 18.75 and 31.25 μmol·m^−2^·s^−1^ groups (*p* < 0.05); at 15 °C, the 18.75 μmol·m^−2^·s^−1^ group yielded the highest chlorophyll a content (9.95 ± 0.30 μg·mL^−1^), significantly exceeding the 0.625 and 31.25 μmol·m^−2^·s^−1^ groups (*p* < 0.05); at 25 °C, the 31.25 μmol·m^−2^·s^−1^ group had the highest chlorophyll a content (12.07 ± 0.97 μg·mL^−1^), significantly higher than the 0.625 and 18.75 μmol·m^−2^·s^−1^ groups (*p* < 0.05). Further simple effects analysis at the optimal light level (31.25 μmol·m^−2^·s^−1^) confirmed that chlorophyll a content at 25 °C was significantly higher than those at 15 °C and 5 °C (*p* < 0.05). Significant interaction effects were also observed for chlorophyll b (F = 59.37, *p* < 0.05) and carotenoids (F = 36.31, *p* < 0.05). Simple effects analysis identified 25 °C + 31.25 μmol·m^−2^·s^−1^ as the optimal preservation conditions, under which chlorophyll b and carotenoid contents reached 8.32 ± 0.84 and 4.34 ± 1.02 μg·mL^−1^, respectively.

In summary, the chlorophyll a, chlorophyll b, and carotenoid contents of all four microalgal strains were all governed by a significant light intensity—temperature interaction, with consistent optimal preservation conditions of 25 °C + 31.25 μmol·m^−2^·s^−1^. In all three strains, the optimal light intensity shifted from low to high as temperature increased, but the mode of this shift was species-specific: *D. abundans* HY-1 exhibited an abrupt crossover (low light was optimal at 5 °C, whereas high light became optimal from 15 °C onward), *C.* sp. displayed a transitional phase at 15 °C in which no significant difference was detected between intermediate and high light, and *O*. sp. HY-1 showed a gradient-like incremental shift from 0.625 to 31.25 μmol·m^−2^·s^−1^. Among the three strains, *D. abundans* HY-1 exhibited the greatest response magnitude (fold-change), whereas *C.* sp. HY-1 attained the highest chlorophyll a content under the optimal conditions (27.12 ± 0.04 μg·mL^−1^).

At the three matched light—temperature pairs (5 °C and 0.625 μmol·m^−2^·s^−1^, 15 °C and 18.75 μmol·m^−2^·s^−1^, and 25 °C and 31.25 μmol·m^−2^·s^−1^), the photosynthetic pigment contents of the control groups (without protective agent) were compared with those of the corresponding groups supplemented with the protective agent after 14 days of preservation. For all four microalgal strains, the chlorophyll a, chlorophyll b, and carotenoid contents in the control groups were significantly lower than those in the matched protective-agent groups at all three conditions (*p* < 0.05). At 25 °C and 31.25 μmol·m^−2^·s^−1^, the chlorophyll a contents of the control groups were 0.22 ± 0.03 μg·mL^−1^ for *D. abundans* HY-1, 3.56 ± 0.14 μg·mL^−1^ for *C*. sp. HY-1, 0.06 ± 0.005 μg·mL^−1^ for *K. cornuta* HY-1, and 0.10 ± 0.01 μg·mL^−1^ for *O*. sp. HY-1, respectively, which were lower than the corresponding protective-agent groups (26.14 ± 0.10, 27.12 ± 0.04, 0.13 ± 0.02, and 12.08 ± 0.97 μg·mL^−1^). The chlorophyll b and carotenoid contents mirrored the response observed for chlorophyll a, whereby the control groups retained significantly lower pigment levels than the matched protective-agent groups across all conditions. In summary, under all three matched light—temperature conditions, the chlorophyll a, chlorophyll b, and carotenoid contents of the control groups (without protective agent) were significantly lower than those of the corresponding protective-agent groups for all four microalgal strains (*p* < 0.05).

## 3. Discussion

The wastewater from a aquaculture recirculation system contains a large amount of nutrients, such as nitrogen and phosphorus. Microalgae can absorb and assimilate these nutrients through photosynthesis. At the same time, numerous studies have shown that cultivating microalgae in wastewater not only can remove a large amount of NH_4_^+^-N, NO_2_-N, TP, and other substances, but also can achieve the growth of microalgae biomass [26,27,28]. This study had preliminarily explored and selected four strains of microalgae (*K. cornuta* HY-1, *D. abundans* HY-1, *C.* sp. HY-1, *O.* sp. HY-1) that are capable of removing nitrogen and phosphorus. A large number of studies have proven that the microalgae selected in this experiment have the ability to remove NH_4_^+^-N [20,29,30]. In this study, the 7-day removal efficiencies of NH_4_^+^-N, NO_2_-N, and TP concentrations in the aquaculture wastewater of the control group (without microalgae) were 5.78 ± 0.61%, 7.00 ± 0.33%, and 5.71 ± 0.05%, respectively. This indicates that aquaculture wastewater is rich in nutrients such as nitrogen and phosphorus. If the participation of photosynthetic autotrophic organisms such as microalgae is lacking, the self-purification capacity of water is significantly insufficient.

In the land-based RASs, all four microalgae have certain removal efficiencies on NH_4_^+^-N, but the efficiencies of the different microalgae vary. The removal efficiency for NH_4_^+^-N by *C.* sp. HY-1 was 87.20 ± 0.56%, significantly higher than those of *D. abundans* HY-1 (56.60 ± 0.44%), *O*. sp. HY-1 (49.33 ± 3.19%), and *K. cornuta* HY-1 (65.30 ± 2.46%) (*p* < 0.05). However, a previous study reported that under the conditions of light intensity (18.75 μmol·m^−2^·s^−1^) and temperature (30 °C), the removal rates for NH_4_^+^-N by *Scenedesmus parvus* and *Coccomyxa dispar* were 65.30% and 56.70%, respectively. The NH_4_^+^-N removal efficiency of *C.* sp. HY-1 in this experiment was significantly higher than the research value in the literature, while *D. abundans* HY-1 was lower than *Scenedesmus parvus* [31]. The reason may be attributed to the differences in the removal efficiencies of different algae under different conditions. Similarly, all four microalgae have removal efficiencies on NO_2_-N, which had also been reported in previous studies [22,32]. After 7 days, *O.* sp. HY-1 had a NO_2_-N removal efficiency of 85.63 ± 2.56%, a removal rate of 0.975 mg·L^−1^·d^−1^. The NO_2_-N removal efficiency of *O*. sp. HY-1 was significantly higher than that of *D. abundans* HY-1 (78.28 ± 0.49%), *K. cornuta* HY-1 (53.36 ± 1.04%), and *C*. sp. HY-1 (69.20 ± 1.42%) (*p* < 0.05). Those indicated that *O.* sp. HY-1 has a significant advantage in purifying NO_2_-N. In contrast, under the conditions of 30 °C and 35 µmol·m^−2^·s^−1^ illumination, the absorption rate for NO_2_-N by *Oocystis borgei* was 0.206 µg·g^−1^·h^−1^ [33]; Mubashar [34] reported that under autotrophic conditions, the removal efficiency for N by *Scenedesmus acuminatus* was 53.57%, which was lower than that of *O*. sp. HY-1 in this study.

Phosphorus is also a common pollutant in the land-based RAS wastewater. All four microalgae in this study have the ability to remove TP, and previous studies also reached a consistent conclusion [20,35,36]. *K. cornuta* HY-1 had a distinct advantage in phosphorus removal efficiency under the tested conditions, achieving 71.19 ± 3.35%. These results demonstrated a clear and prominent advantage of *K. cornuta* HY-1 in TP purification. In comparison, Liu [37] studied, under the conditions of 25 °C and a light intensity of 31.25 μmol·m^−2^·s^−1^, the removal efficiency for TP by *Oocystis borgei* which was lower than that of *K. cornuta* HY-1 in that experiment; Jakhwal et al. [38] found that *T. suecica* achieved high removal efficiencies of TP in recirculating aquaculture systems (RAS) water with low phosphate concentration. Although the microalgae in this study achieved a relatively high removal rate of nitrogen and phosphorus in the wastewater of the recirculating water system, this experiment was conducted under controlled laboratory conditions for wastewater treatment and had not yet been applied to a real recirculating water aquaculture system. In practical applications, there may be fluctuations in wastewater components (such as nitrogen–phosphorus ratio, organic load, and suspended solids concentration), changes in environmental parameters (such as temperature, light intensity, and pH value), and the synergy or competition of biological communities (such as nitrifying bacteria, denitrifying bacteria, and heterotrophic bacteria), which may lead to changes in the actual treatment effect of microalgae. Moreover, this study only conducted a preliminary exploration of the water purification effects of the four microalgae. In the subsequent experiments, further research on the optimal water purification effects of each microalgae and the co-culture water purification mode of the microalgae species will be carried out.

In order to enable microalgae to better resist the adverse effects brought by abiotic stress during large-scale cultivation, this study carried out adaptive laboratory evolution (ALE) of the four microalgae. ALE has been increasingly employed to engineer microalgal strains, with the primary aims of boosting target metabolite productivity and enhancing strain fitness under challenging cultivation conditions [39]. The present study was conducted under controlled laboratory conditions, and previous studies have identified temperature, light intensity, and pH as key limiting factors for large-scale microalgal production [40]. In ALE, selection pressure should be designed following a principle of “staged, progressively intensified” increments. Temperature should be raised by 2–3 °C every 2–4 weeks until the maximum temperature potentially encountered in large-scale production is reached [41]. Light intensity should be increased by 5–10 μmol·m^−2^·s^−1^ every 2–4 weeks until the target high-light level is reached. Starting from the current pH, the value should be adjusted by 0.5–1.0 pH units toward the target direction every 2–4 weeks [39]. Each gradient condition should be maintained in culture until the population growth rate recovers to at least 80% of the control level before advancing to the next step, yielding strains with enhanced tolerance of the microalgae [42]. Furthermore, subsequent experiments may employ whole-genome resequencing, transcriptomic analysis, and physiological phenotyping of the evolved populations to dissect the key genes and metabolic pathways underlying adaptation to temperature, light, pH, and nutrient supply, thereby providing targets for rational genetic engineering.

Microalgae biomass is an important indicator of specific cellular component growth, productivity, and expression [43]. As a core component of photosystem II and photosystem I, chlorophyll a directly participates in the conversion of light energy to chemical energy and is a key substance for microalgae to capture light energy [44,45]. These pigments (carotenoid and chlorophyll b) primarily serve auxiliary energy transfer functions in the photosynthetic system, efficiently transferring absorbed light energy to chlorophyll a while also playing roles in scavenging reactive oxygen species and protecting photosynthetic machinery, ensuring photostability of microalgae under high-light conditions [46]. Under large-scale cultivation conditions, the cell density, biomass dry weight, and photosynthetic pigments of all four strains showed an increasing trend, and they exhibited exponential growth. Previous studies have demonstrated that, under comparable conditions, microalgae growth shows an increasing trend [47,48]. Therefore, the nitrogen and phosphorus in the wastewater from the land-based recirculating water system were absorbed and assimilated via photosynthesis, thereby promoting the growth and reproduction of microalgae. A large number of studies have shown that microalgae can utilize the nitrogen and phosphorus nutrients in the wastewater to convert into their own substances [29,49,50]. Among the four strains, *C*. sp. HY-1 achieved the highest cell density (1.760 ± 0.018 × 10^7^ cells·mL^−1^), whereas *O*. sp. HY-1 accumulated the highest biomass dry weight (223.55 ± 1.47 mg·L^−1^), indicating that these two strains excel in biomass production through different strategies—high cell count and large cell size. Studies had demonstrated that *C*. sp. HY-1 and *O.* sp. HY-1 exhibited the highest biomass accumulation in wastewater aquaculture in the land-based recirculating water system. However, the effect of cell density, cell dry weight, and photosynthetic pigment content was lower than the previous results (2 × 10^7^ cells·mL^−1^, 1 g·L^−1^, and 2 mg·L^−1^) [32,51]. The possible reason might be that the present study did not systematically optimize the growth conditions (temperature, light, pH, nitrogen and phosphorus concentration and ratio, etc.) for the four microalgae.

Growth of the four microalgae reached a stable stage after 14 days of cultivation. To achieve economically efficient microalgae harvesting, chemical flocculants are one of the commonly used methods. This study studied the harvesting effects of inorganic flocculants (iron salts, aluminum salts, and alkali salts) and organic flocculants (chitosan) on the four microalgae, but flocculation efficiency varied significantly with flocculant type and concentration [52,53]. The underlying mechanism is that the algal cells have negative charges due to the presence of functional groups such as carboxyl, phosphate, and hydroxyl groups on their surfaces. The negative charges cause electrostatic repulsion between cells, thus keeping cells in a suspended state. The flocculants are introduced into the cultivation system in ionic form and interact with the negative particles, making the cultivation system unstable, neutralizing the cells, and causing them to flocculate [54].

To elucidate the flocculation mechanisms underlying the observed differences in harvesting efficiency, the present study measured zeta potential and pH under each flocculation condition. Zeta potential reflects the electric potential of the double layer on the particle surface, which determines the electrostatic interaction between particles. When the absolute value of zeta potential is high, the particles in the system carry many negative (positive) charges, and they repel each other [55]. Under such conditions, the system is relatively stable. On the contrary, when zeta potential is close to zero or reverses sign, the electrostatic repulsive force between the particles weakens, promoting the aggregation of particles and the formation of flocs. In the present study, the initial zeta potentials of the four strains ranged from −22.72 to −27.65 mV, confirming their negatively charged surfaces. The surface of microalgae cells is negatively charged due to the dissociation of functional groups such as carboxyl and phosphate groups. Under neutral conditions, zeta potential typically ranges from −20 to −30 mV [56]. The electrostatic repulsion between cells keeps the system dispersed and stable.

We found that when adding strong acid–weak base salts such as aluminum sulfate, potassium aluminum sulfate, FeCl_3_, and FeSO_4_ as flocculants, the H^+^ release from metal ions caused the pH of the system to decrease. Among them, the decrease in trivalent iron salts and aluminum salts is more significant, while ferrous sulfate is more gradua. The decrease in pH not only protonates carboxyl groups on surface of algal cells and reduces the negative charge density, but also promotes the hydrolysis of aluminum and iron ions to form positively charged polymeric hydroxyl complexes and hydroxide precipitates. Through charge neutralization, zeta potential moved towards zero, and when approaching the isoelectric point, flocculation efficiency was the highest [57]. Therefore, the pH change induced by the flocculant was the core intermediate variable connecting the dosage, zeta potential, and flocculation efficiency. Monitoring the changes in pH and zeta potential before and after addition simultaneously can provide a direct basis for determining the optimal dosage of different flocculants. In addition, the flocculation effect of chitosan increased as the concentration increased. The flocculation efficiency of chitosan increased with concentration within the tested range, consistent with previous reports [58,59]. However, chitosan was generally less effective than inorganic flocculants for the four strains examined here. The relatively better performance of chitosan in the present study compared with some previous reports may be attributed to more suitable operating parameters. Excessive addition of chitosan caused charge reversal (positive zeta potentials), which increased electrostatic repulsion between cells and led to restabilization of the suspension, thereby reducing flocculation efficiency [60].

In this study, inorganic flocculants exhibited significantly better effect on microalgae harvesting than organic flocculants. Adding alkali salts (NaOH and Ca(OH)_2_) achieved flocculation by elevating the system pH; Ca(OH)_2_ exhibited significantly higher harvesting efficiency than NaOH. NaOH increased the absolute value of zeta potential, making the system more stable and hindering the flocculation of microalgae. However, at pH > 10.3, the abundant Mg(OH)_2_ precipitates formed a loose network structure that entrapped algal cells via sweep flocculation, resulting in significantly higher flocculation efficiency than that achieved by charge neutralization alone [61]. Therefore, the flocculation efficiency improved with increasing NaOH concentration. Under alkaline conditions, Ca(OH)_2_ precipitates formed in the suspension; these precipitates entrapped microalgal cells via sweep flocculation, while the associated calcium also contributed to charge neutralization, as reflected by zeta potentials approaching zero. It should be noted that although Ca(OH)_2_ is a strong base, its lower aqueous solubility yields a more moderate pH elevation than NaOH at equivalent dosage. During the experiments, the flocculated biomass obtained with Ca(OH)_2_ appeared white, likely due to calcium/magnesium hydroxide precipitates adhering to the cell surface; this residue may affect the subsequent quality and utilization of the harvested biomass [62]. Among the metal coagulants, iron salts (FeCl_3_, FeSO_4_) and aluminum salts (Al_2_(SO_4_)_3_, KAl(SO_4_)_2_) have good harvesting effects on all four types of microalgae. The efficiency of cationic flocculation largely depends on the dosage, with low doses leading to weak bridging and loose flocs, while excessive doses reduce bridging efficiency due to electrostatic repulsion caused by positive charge accumulation [63]. The aluminum salts had the best harvesting effect on *C.* sp. HY-1. Even at a low concentration, they reached over 99%. Moreover, the zeta potentials of 0.5 g·L^−1^ KAl(SO_4_)_2_ and Al_2_(SO_4_)_3_ were 0.73 ± 0.15 and 0.48 ± 0.16 mV, which are consistent with findings reported in previous studies [64]. Previous studies have shown that *Chlorella* sp. had the highest flocculation efficiency at low concentrations of Al_2_(SO_4_)_3_, which is also consistent with our conclusion [56,65,66].

In practical operation, all trivalent ions have considerable flocculation efficiency. Meanwhile, different types of microalgae have a stronger ability to bioaccumulate Fe^3+^ than they do for Fe^2+^ [67]. Among them, the flocculation efficiency of 1.0 g·L^−1^ ferric chloride is higher than those of 0.5 and 1.5 g·L^−1^ [53,68]. In the present study, a low concentration of FeCl_3_ was the most effective for *D. abundans* HY-1, achieving a harvest rate of up to 99.58 ± 0.05% at 0.5 g·L^−1^, with a corresponding zeta potential of −0.61 ± 0.15 mV. The zeta potential of 1.5 g·L^−1^ FeCl_3_ was significantly greater than those of 0.5 and 1.0 g·L^−1^. The flocculation efficiency of 1.5 g·L^−1^ FeCl_3_ was relatively poor, possibly due to the charge reversal (zeta potential shifting from negative to positive values). Excessive Fe^3+^ addition increased electrostatic repulsion between algal cells and reduced flocculation efficiency [69]. Excessive positive ion typically have adverse effects by over-adsorbing positively charged particles onto algal cells, leading to unfavorable electrostatic repulsion and resulting in the re-stabilization of formed flocs [70]. Aluminum salts and iron salts have a good flocculation effect on four types of microalgae, achieving over 90%. However, aluminum exposure leads to the inhibition of the photosynthetic process of microalgae, changes in the cell surface, interference with nutrient absorption, oxidative stress, and a decrease in growth rate, even cell death [71]. Fe^3+^ not only forms hydroxides but also combines with phosphate ions to form precipitates, effectively capturing microalgae in the microalgal precipitates [72]. Based on the obtained results, in the subsequent flocculation process, ferric chloride was selected as the inorganic flocculant, and its performance and low price make it a promising choice.

After harvesting, in order to enable the high-density microalgae to maintain high activity at normal temperatures, this study investigated the effects of light and temperature on the activity of the added microalgae. The results showed that light and temperature both had an impact on the preservation of the four types of algae. There are differences in the rate of photosynthetic pigment loss under different conditions. Studies have shown that algae have the potential to utilize their pigment system as a defense mechanism under various stress conditions [73]. Under all three matched light—temperature conditions, the chlorophyll a, chlorophyll b, and carotenoid contents of the control groups (without protective agent) were significantly lower than those of the corresponding protective-agent groups for all four microalgal strains (*p* < 0.05). Therefore, adding nutrients and protective agents can enhance the microalgal stress resistance, strengthen their cell walls, and improve their survival ability in adverse environments [74]. This study found that the chlorophyll a, chlorophyll b, and carotenoid contents of *D. abundans* HY-1, *C*. sp. HY-1, and *O*. sp. HY-1 were all regulated by significant interactions between light intensity and temperature, with consistent optimal preservation conditions of 25 °C + 31.25 μmol·m^−2^·s^−1^. The contents of photosynthetic pigments in *D. abundans* HY-1, *C.* sp. HY-1, and *O.* sp. HY-1 showed an upward trend, and the contents of photosynthetic pigments in the 25 °C and 31.25 μmol·m^−2^·s^−1^ group were the highest after 14 d, indicating that 25 °C and moderate light supported slow growth and pigment accumulation, thereby minimizing pigment loss during preservation [75]. Under conditions of 25 °C and 31.25 μmol·m^−2^·s^−1^, *D. abundans* HY-1, *C*. sp. HY-1, and *O*. sp. HY-1 had the highest chlorophyll a content (26.13 ± 0.25, 27.12 ± 0.04, and 12.07 ± 0.97 μg·mL^−1^). Guo et al. reported that microalgae preserved at room temperature (25 °C) accumulated photosynthetic pigments more rapidly under higher light intensity, whereas low light was unfavorable for maintaining pigment content [76], consistent with the present results. Other studies have similarly reported increasing pigment trends in microalgae preserved at approximately 25 °C [77].

For *K. cornuta* HY-1, the conditions with the minimum pigment loss among all tested groups were 25 °C and 18.75 μmol·m^−2^·s^−1^ for 14 d. A significant interaction effect of light intensity and temperature was detected for both chlorophyll a and chlorophyll b contents. Under conditions of 25 °C and 18.75 μmol·m^−2^·s^−1^, the photosynthetic pigments of *K. cornuta* HY-1 still showed a downward trend, with loss rates of 67.76% for chlorophyll a, 66.43% for chlorophyll b, and 75.82% for carotenoids. At 25 °C the intermediate irradiance (18.75 μmol·m^−2^·s^−1^) was optimal and high irradiance became inhibitory, consistent with previous reports that excessive light accelerates pigment degradation during microalgal preservation whereas moderate illumination better maintains photosynthetic pigments [78]. In addition, during the room-temperature preservation period, *K. cornuta* HY-1 showed loss of photosynthetic pigments, accompanied by agglomeration and odor, which was similar to the results of related studies [79,80]. The odor likely resulted from bacterial proliferation or decomposition of lysed cellular material, indicating that the protective agent failed to maintain cell integrity and suppress microbial contamination in this strain. The reason may be that this protective agent had no obvious effect on *K. cornuta* HY-1.

However, no significant interaction effect was detected for carotenoids, but a main effect of irradiance was detected (*p* < 0.05), with the lowest content at 0.625 μmol·m^−2^·s^−1^ and no significant difference between 18.75 and 31.25 μmol·m^−2^·s^−1^, indicating a plateau at intermediate-to-high irradiance. This dissociation suggests that carotenoid retention during preservation is primarily light-dependent rather than temperature-dependent, consistent with the photoprotective function of carotenoids [81]; in contrast, chlorophyll a and b appear more strongly modulated by temperature-sensitive processes, likely reflecting their tighter coupling to photosynthetic metabolism. During room temperature preservation, other algae did not show agglomeration or odor, and their color remained bright green, consistent with the results of existing studies [82]. In summary, during the 14-day short-term preservation period, higher light intensity at room temperature (25 °C), in combination with the protective agent, was effective for maintaining and even slowly accumulating photosynthetic pigments in *D. abundans* HY-1, *C*. sp. HY-1, and *O*. sp. HY-1. However, *K. cornuta* HY-1 could not be effectively preserved under any of the tested conditions, and further optimization of the protective agent formulation and preservation parameters is required for this strain.

## 4. Materials and Methods

### 4.1. Microalgae Identification

Water sample (10 mL) was added to sterile BG11 (Qingdao Hope Bio-Technology Co., Ltd., Qingdao, China) [83] liquid culture medium for enrichment. After cultivation, the enriched culture was inoculated onto a BG11 solid medium plate in the ultra-clean workbench (WS-CJ-1FD, Suzhou Purification Equipment Co., Ltd., Suzhou, China) and cultivated under suitable conditions in a light incubator (Jiangnan Light Incubator GXM-258B, Ningbo Jiangnan Instrument Factory, Ningbo, China). When microalgal colonies began to grow, a single algal colony was picked with an inoculation needle, a wet mount was prepared and it was examined under a biological microscope (OLYMPUS BX53, Olympus Co., Ltd., Tokyo, Japan) for screening. Then the target algae were transferred to sterile BG11 liquid medium for further cultivation. After a period of cultivation, a drop of the algal suspension was placed in one depression of a sterile double-depression slide. The position of individual cells was observed, and target cells were isolated using a capillary pipette and transferred to the opposite depression. Purity was verified microscopically. If contamination was detected, this process was repeated until successful separation. A drop of the purified suspension was mounted on a glass slide with a coverslip, immersion oil was added, and the sample was examined under a 100× oil-immersion objective. A 7-day stationary-phase algal culture was harvested, washed twice with sterile water and resuspended in fresh medium containing 2.5 µg·mL^−1^ amoxicillin. The culture was continuously treated for 6–7 days. Subsequently, 1 mL of the algal suspension (approximately 10^2^ to 10^3^ cells) was spread onto nutrient-rich Chytrid Growth Medium [84] solid medium containing the same concentration of amoxicillin. Microalgae were cultivated under the culture conditions for one to two weeks. Single algal colonies were selected without contamination, and were streaked onto modified BG11 [85] solid medium containing amoxicillin for repeated purification. After purification, a single algal colony was inoculated into 100 mL of liquid modified BG11 medium containing amoxicillin for expanded cultivation. The medium was replaced weekly for approximately two months. Ultimately, candidate sterile monoclonal algal strains were obtained [86].

Algal strains were observed under an optical microscope. According to “The Freshwater Algae of China: systematic, Taxonomy and Ecology” [87], identification and classification were carried out based on the morphology of the isolated and purified algal species. Molecular identification was then performed as follows. Approximately 10 mL of algal suspension at the logarithmic growth phase was harvested, and genomic DNA of the microalgae was extracted using a modified CTAB method [88,89]. The ITS universal primers, with the sequence: ITS1: 5′-TCCGTAGGTGAACCTGCGG-3′ and ITS4: 5′-TCCTCCGCTTATTGATATGC-3′, were used for amplification by PCR instrument (TaKaRa TP600, Takara Bio Inc., Kusatsu, Japan). The reaction system (20 μL) consisted of 1 μL DNA template, 1 μL primer ITS1, 1 μL primer ITS4, 10 μL 2 × Taq PCR Master Mix, and 7 μL double-distilled water. The PCR amplification conditions were as follows: 5 min of pre-denaturation at 94 °C, followed by 35 cycles; each cycle included 94 °C denaturation for 30 s, 55 °C annealing for 30 s, and 72 °C extension for 2 min. After the last cycle, the reaction was continued at 72 °C for 5 min, then the products were stored at −20 °C. The PCR amplification products were sent to Sangon Biotech (Shanghai) Co., Ltd., Shanghai, China for sequencing. The full nuclear ribosomal ITS region (ITS1-5.8S-ITS2) of the microalgae was amplified using the primer pair ITS1/ITS4. Bidirectional Sanger reads were assembled into consensus sequences after trimming primers and low-quality regions. The sequences were deposited in GenBank under accession numbers (PZ869733, PZ868274, PZ869735, PZ869736). Processed nucleotide sequences were submitted to BLASTn (https://blast.ncbi.nlm.nih.gov/Blast.cgi) searches against the rRNA/ITS, nr/nt, RefSeq Genome (refseq_genomes), high-throughput genomic sequences (HTGS), and whole-genome shotgun contigs (WGS) databases, including both type and non-type strains. The closest type species were used for identification, with at least 95% sequence similarity cutoff for genus and 99% for species level identification [90].

### 4.2. Evaluation of Nutrient Removal Performance by Microalgae

Aquaculture wastewater with a large amount of nitrogen and phosphorus was sampled from the land-based RASs operated by Hunan Jinshanyuan Agricultural Science and Technology Co., Ltd., Changde, China. To study the N and P removal characteristics of four microalgae in the wastewater of the circulating water system, this study conducted an experiment. The standard analytical methods adopted for water quality detection were specified as follows.

**Figure A1:**
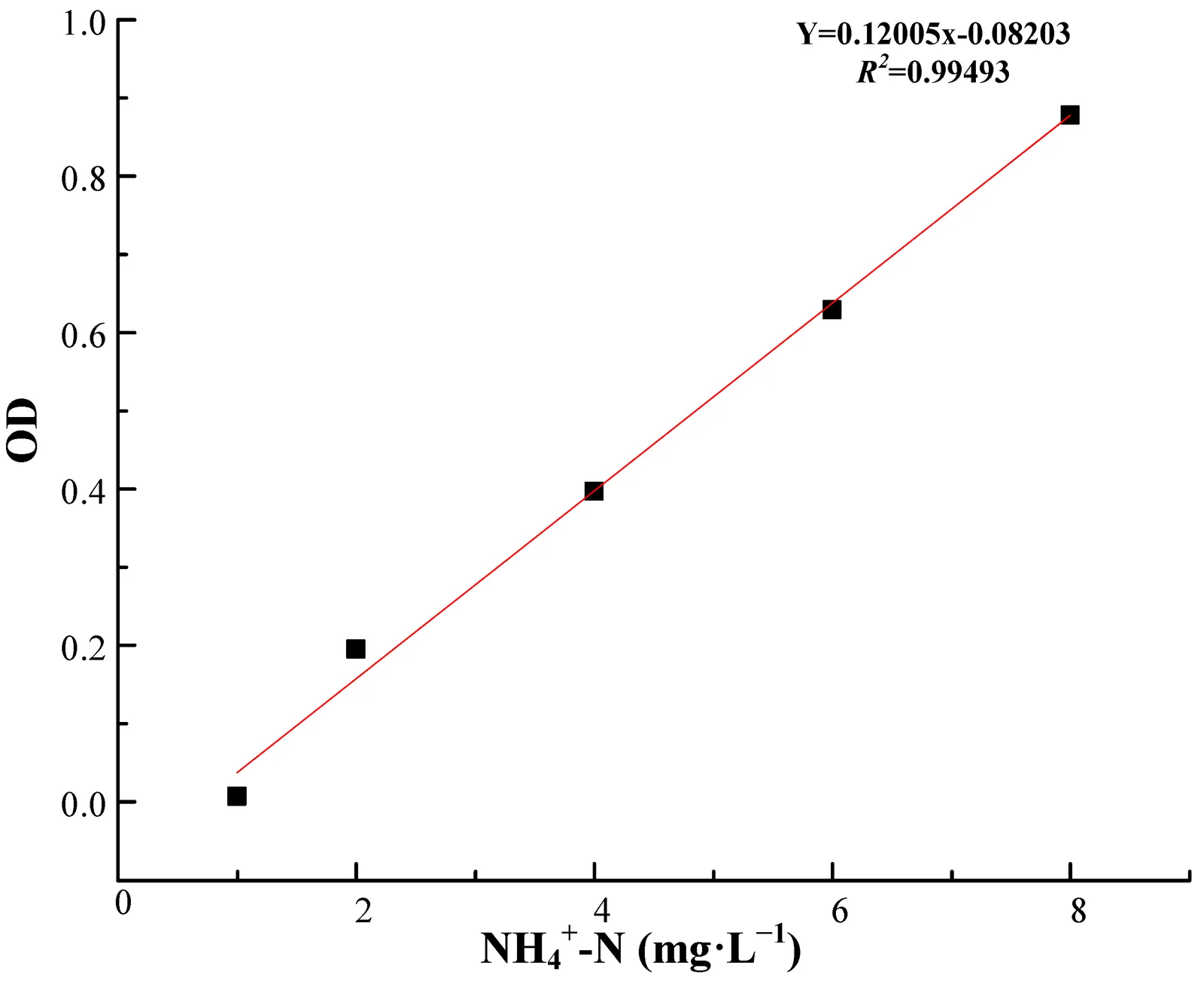
The standard curve of NH_4_^+^-N concentration.

Determination of NH_4_^+^-N in water quality(ISO 7150-1:1984) [91]: Water sample (8.00 mL) was mixed with 1 mL salicylic acid–sodium potassium tartrate reagent, 2 drops of 10 g·L^−1^ sodium nitroprusside, and 2 drops of sodium hypochlorite working solution (available chlorine 3.5 g·L^−1^, free alkali 0.75 mol·L^−1^), then diluted to 10 mL. After 60 min of color development at room temperature, absorbance was measured at 697 nm against water, with a blank run in parallel. A calibration curve was established using a 0–8 μg ammonia nitrogen standard series (1 μg·mL^−1^ as N) processed identically (Figure A1).

**Figure A2:**
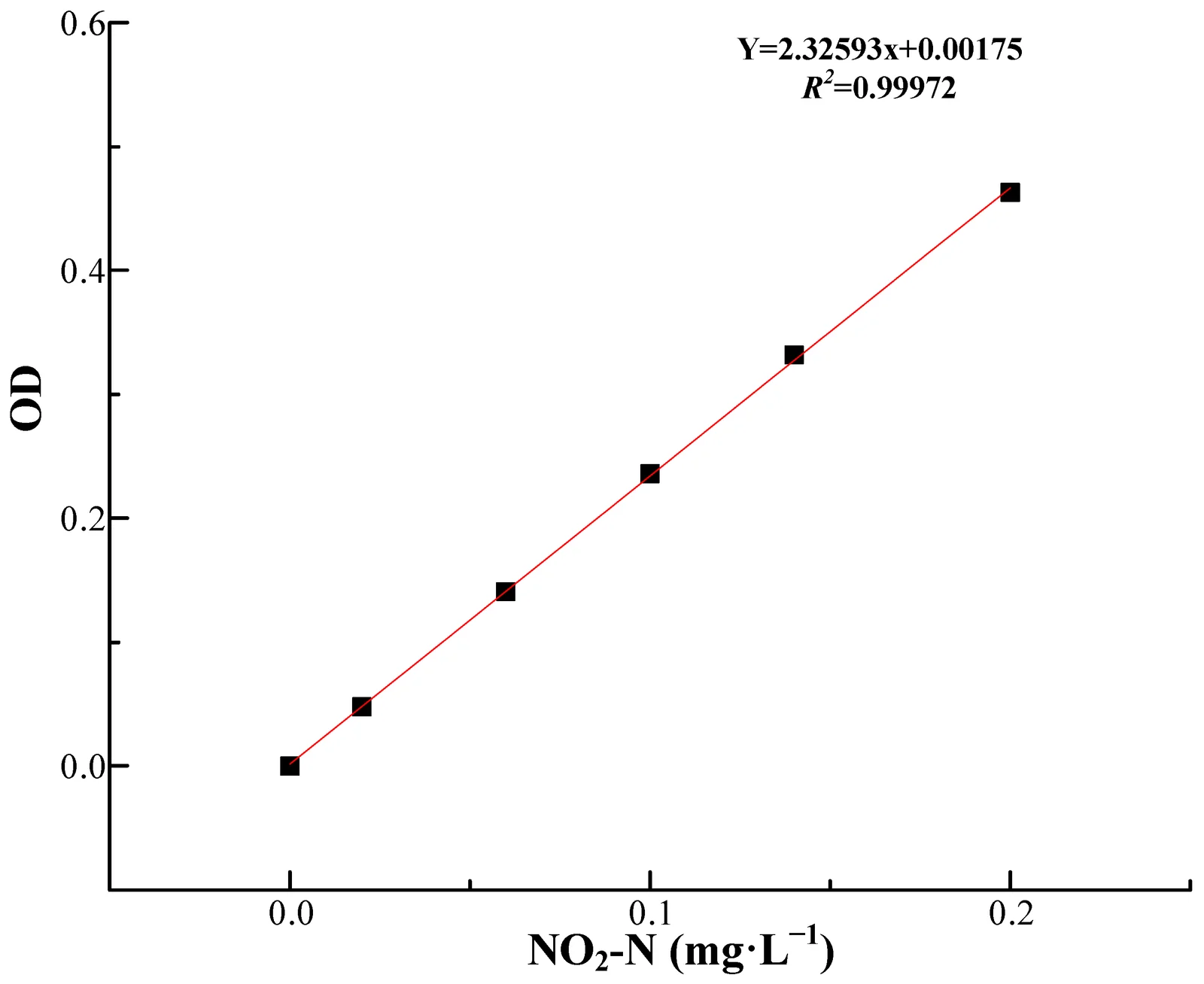
The standard curve of NO_2_-N concentration.

Determination of NO_2_-N in water quality(ISO 6777:1984) [92]: Water sample (50 mL, diluted if necessary) was mixed with 1.0 mL of color reagent containing 4-aminobenzenesulfonamide and N-(1-naphthyl)-ethylenediamine dihydrochloride, and allowed to stand for 20 min to 2 h at pH 1.8 ± 0.3. The absorbance was then measured at 540 nm using a 10 mm cuvette against water, with a blank and color correction run in parallel. A calibration curve was established using a 0–10 μg nitrite nitrogen standard series (1 mg·L^−1^ as N) processed identically (Figure A2).

**Figure A3:**
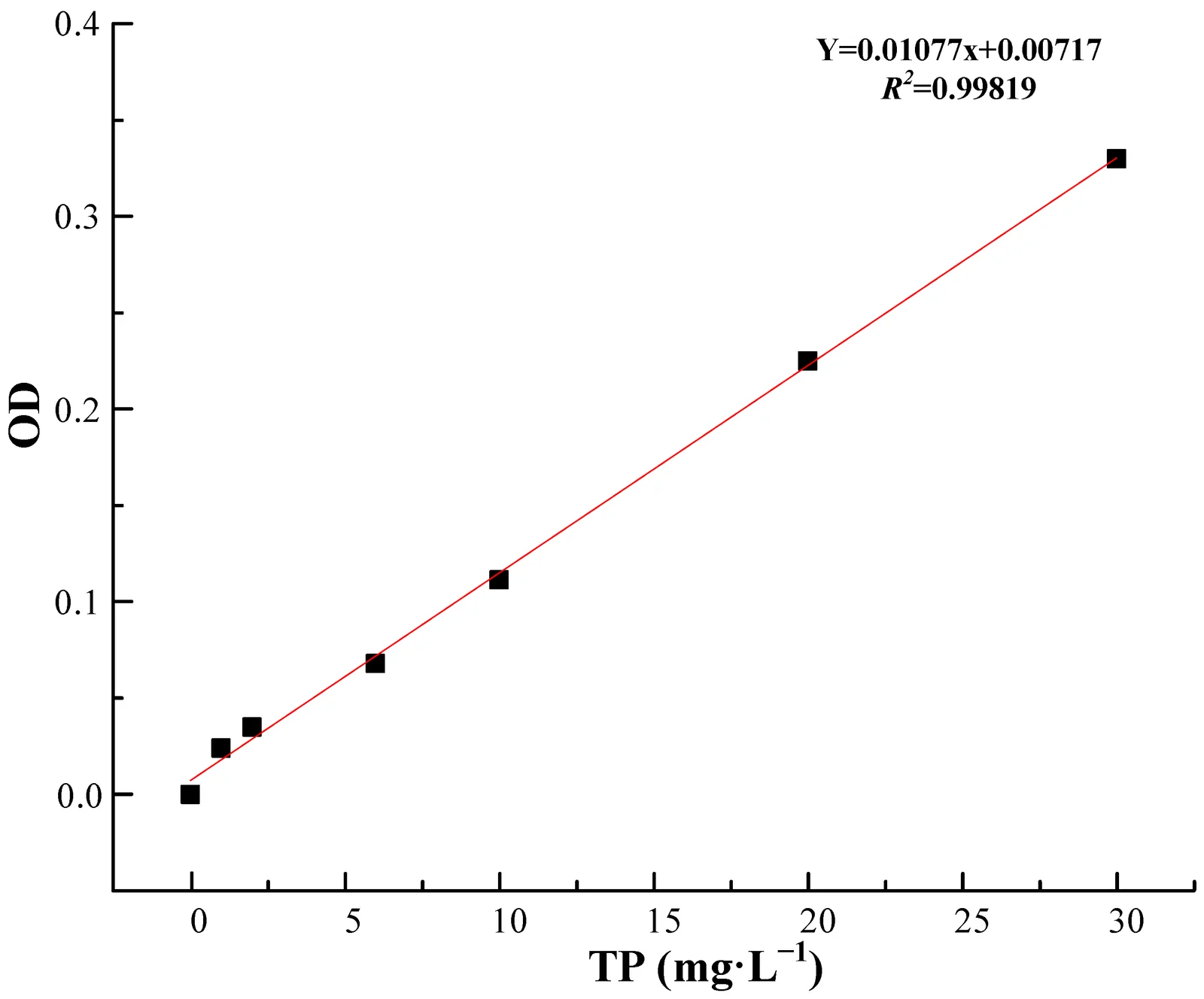
The standard curve of TP concentration.

Determination of TP in water quality (GB 11893-89) [93]: Water sample (25 mL) was digested with 4 mL of 50 g·L^−1^ potassium persulfate in an autoclave at 1.1 kg·cm^−2^ (~120 °C) for 30 min, then cooled and diluted to the mark. After adding 1 mL of 100 g·L^−1^ ascorbic acid and, 30 s later, 2 mL of molybdate reagent (containing ammonium molybdate and potassium antimonyl tartrate), the mixture was allowed to stand for 15 min at room temperature, and the absorbance was measured at 700 nm using a 30 mm cuvette against water, with a blank run in parallel. A calibration curve was established using a 0–30 μg phosphorus standard series (2.0 μg·mL^−1^ as P) processed identically (Figure A3). The tool for determining the OD value was a UV spectrophotometer (UNICO T26 UV-Vis Spectrophotometer, Shanghai Unico Instrument Co., Ltd., Shanghai, China).

### 4.3. Microalgae Large-Scale Cultivation

**Figure 11:**
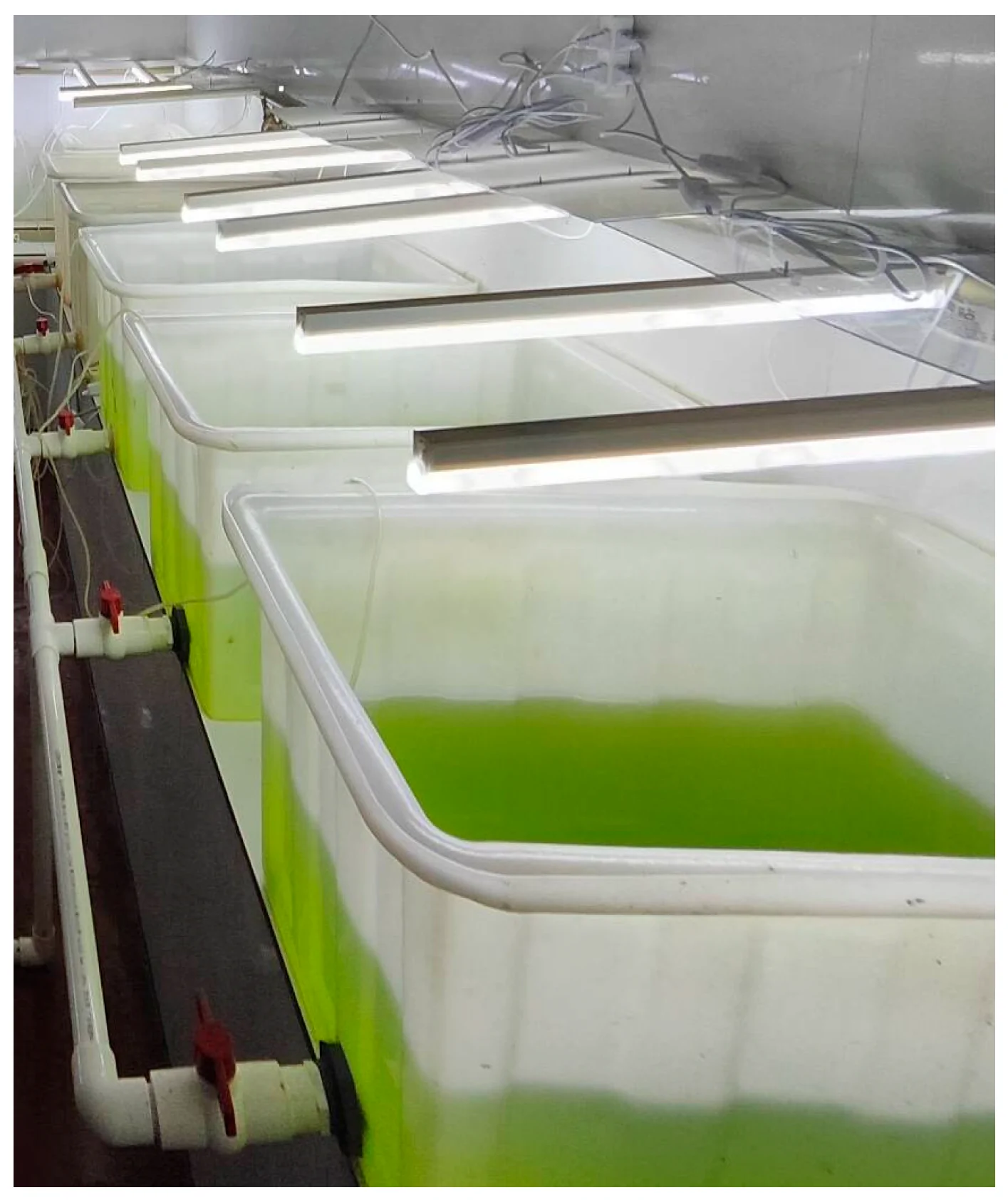
Photobioreactor for microalgae large-scale cultivation.

**Figure A4:**
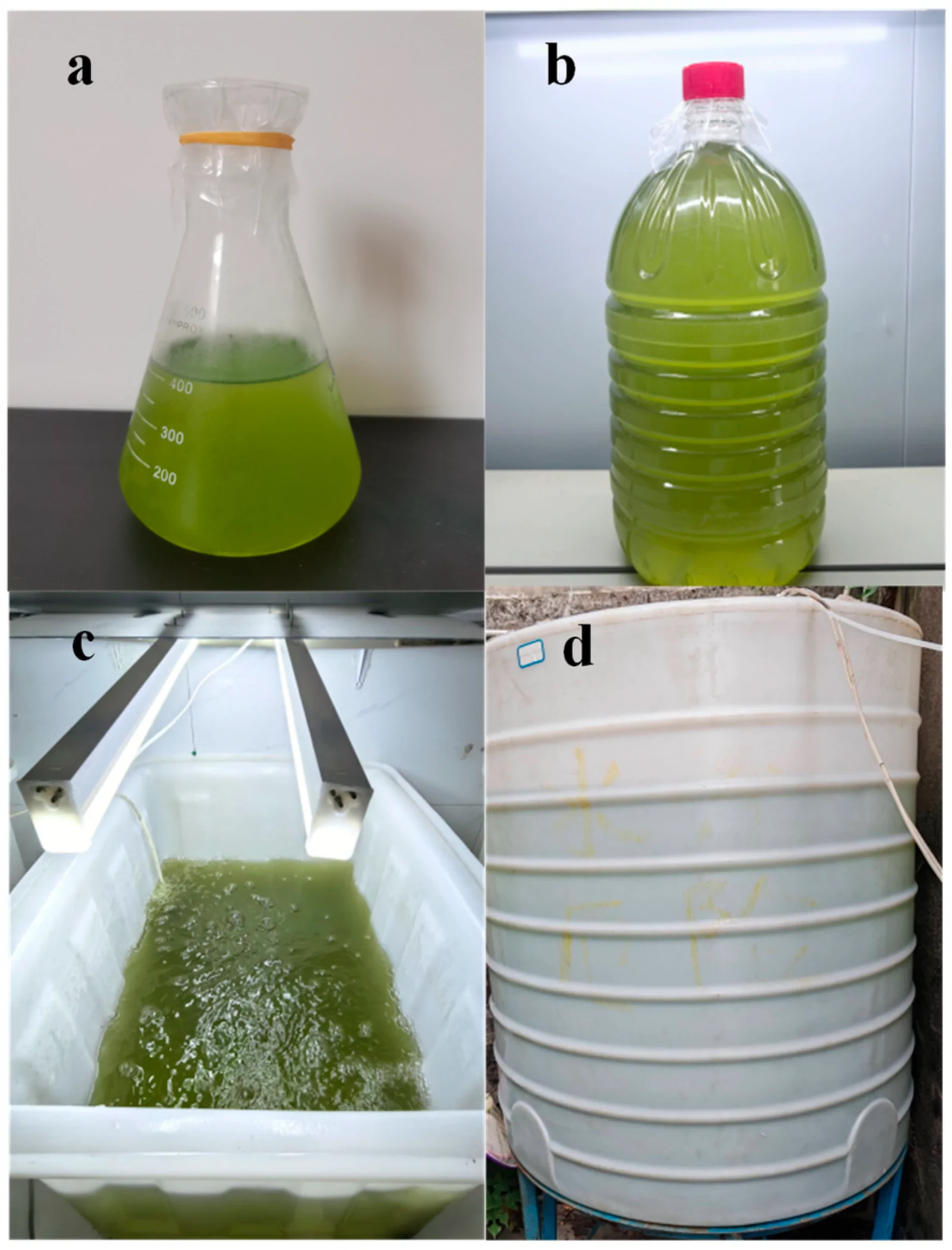
Large-scale cultivation of microalgae. (**a**) Primary culture of microalgae; (**b**) secondary culture of microalgae; (**c**) indoor three-level cultivation of microalgae; (**d**) outdoor three-level cultivation of microalgae.

To systematically evaluate the growth characteristics of *K. cornuta* HY-1, *D. abundans* HY-1, *C*. sp. HY-1, and *O.* sp. HY-1 under practical production conditions, a standardized large-scale cultivation experiment was conducted with strictly controlled environmental parameters. By measuring cell density, dry weight and pigments, the growth status of microalgae during large-scale culture was described from both macroscopic and microscopic perspectives (Figure 11 and Figure A4).

#### 4.3.1. Measurement of Cell Density and Biomass

Cell density was quantified using a hemocytometer. An aliquot of homogenized algal suspension was loaded onto both counting chambers of the hemocytometer, covered with a coverslip, and left to settle for 10 min. The suspension was then observed under an optical microscope. Initially, the grid network was located using a low-power lens, followed by switching to a high-power lens to count the cells in the four corners (upper right, lower right, upper left, lower left) and the center of each small grid. Counting was repeated three times, and the data were recorded. The number of algal cells per milliliter of suspension was calculated using the following formula [94].

*C* = *N*/*n* × 25 × 10,000 × *f* (1)

*C*: Cell density (cells·mL^−1^).

*N*: Count the total number of cells.

*n*: Number of squares counted.

*f*: Dilution ratio.

Quantitative filter paper was pre-dried to constant weight at 80 °C in a hot-air drying oven (WGL-125L, Tianjin Test Instrument Co., Ltd., Tianjin, China) and weighed *M*_0_. A 30 mL algal aliquot was then vacuum-filtered through the pre-dried filter paper until the filtrate was clear. The filter paper with algal cells was further dried to constant weight and weighed *M*_1_. Three replicate groups were set up, and the average value was taken. The dry weight (*DW*) of the algal cells was then calculated using the following formula [95]:

*DW* = (*M*_1_ − *M*_0_)/30 × 1000 (2)

*DW*: Microalgae dry weight, mg·L^−1^.

*M*_0_: Blank membrane filter constant weight, g.

*M*_1_: Algae membrane filter constant weight, g.

Specific growth rate:

*µ* = (ln*X_t_*_2_ − ln*X_t_*_1_)/(*t*_2_ − *t*_1_) (3)

*µ*: Specific growth rate, d^−1^.

*X_t_*_2_: At time t_2,_ the microalgae cell density (cells·mL^−^^1^) or the dry weight of microalgae cells (mg·L^−^^1^).

*X_t_*_1_: At time t_1_, the microalgae cell density (cells·mL^−^^1^) or the dry weight of microalgae cells (mg·L^−^^1^).

Absolute instantaneous velocity

*V*(*x*) = d*y*/d*x* (4)

*y*: The biomass at the culture time *x* (d).

*V*(*x*): The instantaneous absolute growth rate at Day *x*, μg·mL^−1^·d^−1^.

#### 4.3.2. Chlorophyll a, Chlorophyll b, and Carotenoids Content Determination

Photosynthetic pigment contents were characterized using ultraviolet–visible absorption spectroscopy. A total of 10 mL of algal suspension was filtered through a 0.45 μm microporous membrane under a vacuum pressure of 0.02–0.05 MPa. The filter membrane was then cut into pieces and placed in a test tube containing 10 mL of 95% ethanol solution. The test tube was placed in a 75 °C constant-temperature water bath for 15 min. The solution was then transferred to a 10 mL centrifuge tube and centrifuged at 4000 rpm for 15 min using a centrifuge (Eppendorf 5415R Micro high-speed refrigerated centrifuge, Eppendorf, Hamburg, Germany). The supernatant was collected, and the absorbance values at 665 nm, 649 nm, and 470 nm were measured using a UV–visible spectrophotometer. Chlorophyll content was calculated using the following formulas [83]:

*Chlorophyll a* (μg·mL^−1^) = 13.36 *A*_665_ − 5.19 *A*_649_

*Chlorophyll b* (μg·mL^−1^) = 27.43 *A*_649_ − 8.12 *A*_665_

*Carotenoids* (μg·mL^−1^) = (1000*A*_470_ − 2.13*C_a_* − 97.64*C_b_*)/209 (5)

*A*_665_: Absorbance of the extract at 665 nm.

*A*_649_: Absorbance of the extract at 649 nm.

*A*_470_: Absorbance of the extract at 470 nm.

### 4.4. Microalgae Harvesting

#### 4.4.1. Microalgae Chemical Flocculation

Based on the growth curve and OD value of the algal suspension [84], 100 mL of algal suspension in the logarithmic growth phase was taken and placed in a 100 mL beaker. A certain concentration of flocculant was added, and the mixture was stirred rapidly with a glass rod for 2 min and then allowed to stand for a specified time. After standing, an appropriate amount of algal suspension was aspirated from the middle of the solution at the same position using a pipette, and the absorbance value was measured. The position of aspiration was kept unchanged, and the movement was as gentle as possible to avoid the floating of already flocculated algal bodies. The absorbance value at 680 nm (OD_680nm_) was measured, and the flocculation efficiency (%) was calculated using the following formula:

*R* = (*OD_t_*_0_ − *OD_t_*)/*OD_t_*_0_ × 100% (6)

*R*: Removal efficiency, %.

*OD_t_*_0_: Initial absorbance value of the algal suspension.

*OD_t_*: Absorbance value of the algal suspension at time.

#### 4.4.2. Zeta Potential and pH Measurement

The zeta potential reflected the charge status on the surface of microbial cells. By measuring the zeta potential of microorganisms and carriers under different conditions, the electrostatic attractive or repulsive force between them was analyzed, thereby explaining the flocculation effect of flocculants on microalgae. After adding the flocculant, the liquid was left to stand for 145 min. Zeta potential of the supernatant was measured by a zeta potential analyzer (NanoBrook 90S PALS, Brookhaven Instruments Corporation, Nashua, NH, USA) at room temperature of 20 ± 2 °C [66]. The pH values of the supernatant were determined by a pH meter (pH meter PHS-3C, Shanghai INESA Scientific Instrument Co., Ltd., Shanghai, China).

### 4.5. Preservation at Room Temperature

The concentrated algal liquid was obtained through flocculation with ferric chloride. To ensure the standardization of the initial biomass of each group, the initial densities of the four microalgae were all 6.8 × 10^7^ cells·mL^−1^. A nutrient formula and protective agent (protective agent formula: 50 mg·L^−1^ NaNO_3_, 5 mg·L^−1^ K_2_HPO_4_, 0.2 mg·L^−1^ FeC_6_H_5_O_7_, 0.30 g·L^−1^ NaHCO_3_, 120 μg·L^−1^ vitamin B_1_, 0.30 μg·L^−1^ vitamin B_12_) [74] were added, and the mixture was thoroughly homogenized. The experiment was conducted to assess the effects of temperature, light intensity, and protective agent supplementation (present vs. absent) on the physiological stability of the four strains during a 14-day preservation period. Chlorophyll concentrations were determined every two days throughout the preservation trial to monitor algal physiological status [82,96].

### 4.6. Experimental Design and Statistical Analysis

The entire study comprised four experimental modules, each addressing a distinct stage of development of microalgae products based on wastewater treatment: nitrogen and phosphorus removal; large-scale cultivation; flocculation harvesting; and short-term preservation.

Firstly, to explore the feasibility of using microalgae biotechnology for in situ wastewater treatment, four species of microalgae and a blank control were set up to treat the collected wastewater. The entire cultivation system was maintained at a stable temperature of (25 ± 1) °C, pH 7.00 to 7.60, and a light intensity of (37.5 ± 1.25) μmol·m^−2^·s^−1^ (12L:12D) in a light incubator. The initial concentrations of NH_4_^+^-N, NO_2_-N, and TP in aquaculture wastewater were 0.919 ± 0.008, 7.820 ± 0.107, and 1.502 ± 0.020 mg·L^−1^, respectively. Water samples were collected daily to determine residual NH_4_^+^-N, NO_2_-N, and TP concentrations.

Secondly, this section aimed to understand the growth characteristics of microalgae during in situ wastewater treatment, so the culture solution used was still in situ wastewater. Under the same culture conditions mentioned above, growth kinetics of the four strains were monitored in the photoreactor (50 L) over a 14-day period. Samples were collected at 2-day intervals to determine cell density, biomass dry weight, and photosynthetic pigment (chlorophyll a, chlorophyll b, and carotenoid) contents.

Thirdly, a single-factor experiment was designed to optimize the chemical harvesting of the four strains. Seven flocculants were evaluated: chitosan, Ca(OH)_2_, NaOH, KAl(SO_4_)_2_, Al_2_(SO_4_)_3_, FeCl_3_, and FeSO_4_, each at three concentration levels (0.5, 1.0, and 1.5 g·L^−1^). Flocculation efficiency was monitored at 5, 35, 65, 95, and 145 min after flocculant addition. Concurrently, zeta potential and pH were measured for each treatment to elucidate the underlying flocculation mechanism.

Finally, a experiment was set up with two factors: light intensity (0.625 μmol·m^−2^·s^−1^, 18.75 μmol·m^−2^·s^−1^, 31.25 μmol·m^−2^·s^−1^) and temperature (5 °C, 15 °C, 25 °C). All nine treatment combinations were supplemented with protective agent. Moreover, to preliminarily verify protective efficacy under representative low-, medium- and high- stress conditions, three control groups without the agent were established at matched temperature–light levels: low-low (5 °C + 0.625 μmol·m^−2^·s^−1^), medium–medium (15 °C + 18.75 μmol·m^−2^·s^−1^), and high–high (25 °C + 31.25 μmol·m^−2^·s^−1^). Independent-samples *t*-tests with Bonferroni correction were conducted between each matched pair. The concentrated algal suspension was placed in an intelligent incubator (GXM-258B) for 14 days, with photosynthetic pigment (chlorophyll a, chlorophyll b, and carotenoid) contents measured at 2-day intervals.

All data are expressed as mean ± SD of three independent biological replicates. The temporal trends of cell density and dry weight during large-scale cultivation were fitted using the exponential model *f*(*x*) = e^(a+b*x*)^, and photosynthetic pigment content was fitted using the modified exponential model *f*(*x*) = *x*_0_ + b·e^(ax)^, where *x* is cultivation time. Differences in NH_4_^+^-N, NO_2_-N, and TP removal efficiencies, biomass concentration, flocculation efficiencies, zeta potential, and photosynthetic pigment contents were analyzed by one-way ANOVA followed by a Tukey test. Before the analysis of variance, zeta potential value was converted to absolute values to eliminate the directional interference caused by positive and negative charges, ensuring that the comparison between groups only reflects the difference in charge magnitude, rather than the difference in charge sign. A significance threshold of *p* < 0.05 was applied throughout. All statistical analyses were conducted using IBM SPSS Statistics 27.0. Figures were generated using Origin 2024.

## 5. Conclusions

In this study, four microalgal species with removal effects on ammonia nitrogen (NH_4_^+^-N), nitrite nitrogen (NO_2_-N) and total phosphorus (TP) were isolated and identified from the land-based RASs, namely, *Kirchneriella cornuta* HY-1, *Desmodesmus abundans* HY-1, *Coccomyxa* sp. HY-1 and *Oocystis* sp. HY-1. Under large-scale culture conditions of pH 7.00–7.60, (25 ± 1) °C, and light intensity (37.5 ± 1.25) μmol·m^−2^·s^−1^ (12L:12D), the harvest period could be reached in in situ wastewater after about 14 days of cultivation. The optimal flocculant was 1.0 g·L^−1^ FeCl_3_ for harvesting. With the addition of a nutrient formula and protective agent, the optimal preservation conditions for *D. abundans* HY-1, *C.* sp. HY-1, and *O.* sp. HY-1 were 25 °C and 31.25 μmol·m^−2^·s^−1^, while for *K. cornuta* HY-1 they were 25 °C and 18.75 μmol·m^−2^·s^−1^. These findings provided the key technologies including cultivation, harvesting, and preservation for the identified microalgal large-scale production. Moreover, they also verified the technical practicability of in situ wastewater treatment via microalgae-based biotechnology.

## Figures and Tables

**Table 1:** Taxonomic assignment of ITS 1, 5.8S ribosomal RNA gene, and ITS 2 sequences deriving from Chlorophyta isolates.

Strain (Accession Number)	Origin	Identification	Closest Species	Accession Number	Similarity (%)	Taxonomy
HYNU202601 (GenBank: PZ869733)	Land-based recirculating aquaculture systems, Hengyang	*Kirchneriella cornuta* HY-1	*Kirchneriella cornuta* CFR 8-01/FW	KJ680139.1 (GenBank)	99.29%	p_Chlorophyta; c_Chlorophyceae; o_Sphaeropleales; f_Selenastraceae; g_*Kirchneriella*
HYNU202602 (GenBank: PZ868274)	Land-based recirculating aquaculture systems, Hengyang	*Desmodesmus abundans* HY-1	*Desmodesmus abundans* TAU-MAC 3110	MK496927.1 (GenBank)	100%	p_Chlorophyta; c_Chlorophyceae; o_Sphaeropleales; f_Scenedesmaceae; g_*Desmodesmus*
HYNU202603 (GenBank: PZ869735)	Land-based recirculating aquaculture systems, Hengyang	*Coccomyxa* sp. HY-1	*Coccomyxa* sp. LXD	KR149246.1 (GenBank)	99.52%	p_Chlorophyta; c_Trebouxiophyceae; o_Trebouxiophyceae incertae sedis; f_Coccomyxaceae; g_*Coccomyxa*
HYNU202604 (GenBank: PZ869736)	Land-based recirculating aquaculture systems, Hengyang	*Oocystis* sp. HY-1	*Oocystis* sp. CAUP H1110	KY038331.1 (GenBank)	100%	p_Chlorophyta; c_Trebouxiophyceae; o_Chlorellales; f_Oocystaceae; g_*Oocystis*

Abbreviations: p: phylum; c: class; o: order; f: family; g: genus.

**Table 2:** The removal efficiency for NH_4_^+^-N by four microalgae species.

Time	NH_4_^+^-N Concentration in Wastewater (mg·L^−1^)				
	Control	*K. cornuta* HY-1	*D. abundans* HY-1	*C*. sp. HY-1	*O*. sp. HY-1
D1	0.917 ± 0.004 ^a^	0.918 ± 0.020 ^a^	0.909 ± 0.003 ^a^	0.933 ± 0.032 ^a^	0.920 ± 0.016 ^a^
D2	0.912 ± 0.003 ^d^	0.806 ± 0.004 ^b^	0.873 ± 0.019 ^c^	0.808 ± 0.002 ^b^	0.757 ± 0.005 ^a^
D3	0.904 ± 0.002 ^c^	0.694 ± 0.007 ^a^	0.787 ± 0.007 ^b^	0.689 ± 0.015 ^a^	0.704 ± 0.007 ^a^
D4	0.892 ± 0.003 ^e^	0.577 ± 0.002 ^b^	0.710 ± 0.013 ^d^	0.497 ± 0.020 ^a^	0.641 ± 0.015 ^c^
D5	0.881 ± 0.003 ^d^	0.422 ± 0.024 ^b^	0.595 ± 0.005 ^c^	0.293 ± 0.007 ^a^	0.581 ± 0.005 ^c^
D6	0.870 ± 0.003 ^e^	0.357 ± 0.008 ^b^	0.475 ± 0.008 ^c^	0.242 ± 0.007 ^a^	0.503 ± 0.009 ^d^
D7	0.864 ± 0.002 ^e^	0.318 ± 0.016 ^b^	0.395 ± 0.005 ^c^	0.120 ± 0.007 ^a^	0.466 ± 0.023 ^d^

Note: One-way ANOVA was performed for each day. Different superscript letters within the same row indicate significant differences (*p* < 0.05).

**Table 3:** The removal efficiency for NO_2_-N by four microalgae species.

Time	NO_2_-N Concentration in Wastewater (mg·L^−1^)				
	Control	*K. cornuta* HY-1	*D. abundans* HY-1	*C.* sp. HY-1	*O.* sp. HY-1
D1	7.843 ± 0.011 ^a^	7.640 ± 0.044 ^a^	7.803 ± 0.156 ^a^	7.837 ± 0.049 ^a^	7.975 ± 0.158 ^a^
D2	7.762 ± 0.017 ^d^	6.945 ± 0.075 ^b^	6.002 ± 0.173 ^a^	7.125 ± 0.067 ^bc^	7.450 ± 0.151 ^c^
D3	7.662 ± 0.016 ^d^	6.390 ± 0.315 ^bc^	5.251 ± 0.032 ^a^	5.928 ± 0.238 ^b^	6.543 ± 0.010 ^c^
D4	7.537 ± 0.029 ^c^	5.108 ± 0.330 ^b^	3.977 ± 0.093 ^a^	5.230 ± 0.269 ^b^	5.761 ± 0.087 ^b^
D5	7.428 ± 0.017 ^c^	4.672 ± 0.360 ^b^	3.026 ± 0.016 ^a^	3.510 ± 0.106 ^a^	3.027 ± 0.040 ^a^
D6	7.365 ± 0.013 ^d^	4.220 ± 0.239 ^c^	1.924 ± 0.020 ^a^	2.883 ± 0.109 ^b^	1.855 ± 0.133 ^a^
D7	7.294 ± 0.017 ^e^	3.564 ± 0.097 ^d^	1.695 ± 0.057 ^b^	2.414 ± 0.122 ^c^	1.146 ± 0.22 ^a^

Note: One-way ANOVA was performed for each day. Different superscript letters within the same row indicate significant differences (*p* < 0.05).

**Table 4:** The removal efficiency for TP by four microalgae species.

Time	TP Concentration in Wastewater (mg·L^−1^)				
	Control	*K. cornuta* HY-1	*D. abundans* HY-1	*C.* sp. HY-1	*O.* sp. HY-1
D1	1.505 ± 0.001 ^a^	1.501 ± 0.006 ^a^	1.469 ± 0.018 ^a^	1.500 ± 0.044 ^a^	1.533 ± 0.125 ^a^
D2	1.487 ± 0.001 ^c^	1.245 ± 0.002 ^ab^	1.351 ± 0.006 ^b^	1.317 ± 0.013 ^ab^	1.220 ± 0.087 ^a^
D3	1.465 ± 0.001 ^c^	1.090 ± 0.046 ^a^	1.202 ± 0.026 ^b^	1.220 ± 0.057 ^b^	1.047 ± 0.043 ^ab^
D4	1.437 ± 0.001 ^c^	0.955 ± 0.092 ^ab^	1.053 ± 0.012 ^b^	1.082 ± 0.004 ^b^	0.905 ± 0.006 ^a^
D5	1.432 ± 0.001 ^c^	0.757 ± 0.033 ^a^	0.785 ± 0.000 ^a^	0.850 ± 0.008 ^b^	0.807 ± 0.009 ^a^
D6	1.423 ± 0.001 ^d^	0.564 ± 0.003 ^a^	0.689 ± 0.009 ^b^	0.789 ± 0.020 ^c^	0.648 ± 0.024 ^b^
D7	1.419 ± 0.002 ^d^	0.433 ± 0.052 ^a^	0.645 ± 0.010 ^b^	0.730 ± 0.021 ^c^	0.603 ± 0.014 ^b^

Note: One-way ANOVA was performed for each day. Different superscript letters within the same row indicate significant differences (*p* < 0.05).

**Table 5:** The changes in chlorophyll a content of four species of algae during the 14-day culture process.

Time	Chlorophyll a Content (μg·mL^−1^)			
	*K. cornuta* HY-1	*D. abundans* HY-1	*C.* sp. HY-1	*O.* sp. HY-1
D1	0.089 ± 0.028 ^ab^	0.152 ± 0.031 ^b^	0.156 ± 0.013 ^b^	0.051 ± 0.011 ^a^
D2	0.113 ± 0.027 ^a^	0.176 ± 0.014 ^a^	0.290 ± 0.047 ^b^	0.143 ± 0.010 ^a^
D3	0.151 ± 0.034 ^a^	0.187 ± 0.042 ^ab^	0.352 ± 0.089 ^b^	0.166 ± 0.019 ^a^
D4	0.161 ± 0.023 ^a^	0.215 ± 0.049 ^a^	0.511 ± 0.043 ^b^	0.184 ± 0.021 ^a^
D5	0.170 ± 0.026 ^a^	0.234 ± 0.056 ^a^	0.657 ± 0.049 ^b^	0.220 ± 0.028 ^a^
D6	0.204 ± 0.028 ^a^	0.276 ± 0.060 ^a^	0.783 ± 0.017 ^b^	0.250 ± 0.012 ^a^
D7	0.231 ± 0.026 ^a^	0.307 ± 0.081 ^a^	0.870 ± 0.09 ^b^	0.300 ± 0.016 ^a^
D8	0.271 ± 0.011 ^a^	0.348 ± 0.094 ^a^	0.928 ± 0.093 ^b^	0.325 ± 0.021 ^a^
D9	0.302 ± 0.008 ^a^	0.411 ± 0.114 ^a^	0.996 ± 0.080 ^b^	0.377 ± 0.011 ^a^
D10	0.343 ± 0.020 ^a^	0.435 ± 0.112 ^a^	1.045 ± 0.078 ^b^	0.427 ± 0.021 ^a^
D11	0.361 ± 0.019 ^a^	0.500 ± 0.090 ^a^	1.178 ± 0.096 ^b^	0.471 ± 0.016 ^a^
D12	0.386 ± 0.027 ^a^	0.531 ± 0.078 ^a^	1.293 ± 0.034 ^b^	0.494 ± 0.015 ^a^
D13	0.419 ± 0.038 ^b^	0.578 ± 0.082 ^a^	1.398 ± 0.019 ^c^	0.523 ± 0.004 ^b^
D14	0.422 ± 0.027 ^a^	0.598 ± 0.086 ^b^	1.482 ± 0.020 ^c^	0.547 ± 0.012 ^a^

One-way ANOVA was performed for each day. Different superscript letters within the same row indicate significant differences (*p* < 0.05).

**Table 6:** The changes in chlorophyll b content of four species of algae during the 14-day culture process.

Time	Chlorophyll b Content (μg·mL^−1^)			
	*K. cornuta* HY-1	*D. abundans* HY-1	*C.* sp. HY-1	*O.* sp. HY-1
D1	0.051 ± 0.022 ^a^	0.134 ± 0.056 ^b^	0.237 ± 0.019 ^c^	0.042 ± 0.007 ^a^
D2	0.074 ± 0.036 ^a^	0.192 ± 0.014 ^b^	0.261 ± 0.026 ^b^	0.099 ± 0.024 ^a^
D3	0.078 ± 0.033 ^a^	0.218 ± 0.022 ^ab^	0.263 ± 0.081 ^b^	0.142 ± 0.010 ^ab^
D4	0.129 ± 0.012 ^a^	0.258 ± 0.023 ^bc^	0.321 ± 0.053 ^c^	0.171 ± 0.019 ^ab^
D5	0.166 ± 0.015 ^a^	0.255 ± 0.013 ^b^	0.442 ± 0.031 ^c^	0.215 ± 0.026 ^a^
D6	0.181 ± 0.018 ^a^	0.309 ± 0.033 ^b^	0.527 ± 0.058 ^c^	0.261 ± 0.032 ^ab^
D7	0.216 ± 0.020 ^a^	0.355 ± 0.037 ^b^	0.628 ± 0.037 ^c^	0.311 ± 0.043 ^ab^
D8	0.231 ± 0.023 ^a^	0.387 ± 0.043 ^a^	0.698 ± 0.091 ^b^	0.345 ± 0.043 ^a^
D9	0.237 ± 0.018 ^a^	0.430 ± 0.061 ^a^	0.714 ± 0.115 ^b^	0.362 ± 0.025 ^a^
D10	0.294 ± 0.048 ^a^	0.472 ± 0.024 ^a^	0.829 ± 0.107 ^b^	0.388 ± 0.019 ^a^
D11	0.323 ± 0.062 ^a^	0.481 ± 0.035 ^b^	0.878 ± 0.142 ^c^	0.426 ± 0.021 ^a^
D12	0.356 ± 0.068 ^a^	0.527 ± 0.053 ^b^	0.938 ± 0.104 ^c^	0.452 ± 0.014 ^a^
D13	0.368 ± 0.054 ^a^	0.571 ± 0.041 ^a^	0.987 ± 0.153 ^b^	0.483 ± 0.022 ^a^
D14	0.415 ± 0.077 ^a^	0.607 ± 0.059 ^a^	1.082 ± 0.222 ^b^	0.485 ± 0.027 ^a^

One-way ANOVA was performed for each day. Different superscript letters within the same row indicate significant differences (*p* < 0.05).

**Table 7:** The changes in carotenoid content of four species of algae during the 14-day culture process.

Time	Carotenoid Content (μg·mL^−1^)			
	*K. cornuta* HY-1	*D. abundans* HY-1	*C.* sp. HY-1	*O.* sp. HY-1
D1	0.057 ± 0.014 ^b^	0.039 ± 0.012 ^ab^	0.043 ± 0.012 ^ab^	0.012 ± 0.005 ^a^
D2	0.058 ± 0.018 ^a^	0.042 ± 0.00 ^a^	0.086 ± 0.027 ^a^	0.032 ± 0.011 ^a^
D3	0.066 ± 0.016 ^a^	0.057 ± 0.006 ^a^	0.093 ± 0.028 ^a^	0.044 ± 0.006 ^a^
D4	0.063 ± 0.015 ^a^	0.066 ± 0.007 ^a^	0.143 ± 0.003 ^b^	0.051 ± 0.007 ^a^
D5	0.070 ± 0.017 ^a^	0.081 ± 0.017 ^a^	0.202 ± 0.034 ^b^	0.063 ± 0.015 ^a^
D6	0.079 ± 0.013 ^a^	0.098 ± 0.020 ^a^	0.268 ± 0.018 ^b^	0.058 ± 0.014 ^a^
D7	0.084 ± 0.009 ^ab^	0.107 ± 0.016 ^b^	0.320 ± 0.010 ^c^	0.056 ± 0.016 ^a^
D8	0.093 ± 0.015 ^ab^	0.123 ± 0.019 ^b^	0.362 ± 0.020 ^c^	0.062 ± 0.015 ^a^
D9	0.113 ± 0.022 ^a^	0.133 ± 0.022 ^a^	0.397 ± 0.035 ^b^	0.079 ± 0.014 ^a^
D10	0.118 ± 0.022 ^a^	0.146 ± 0.026 ^a^	0.428 ± 0.032 ^b^	0.111 ± 0.024 ^a^
D11	0.142 ± 0.016 ^a^	0.153 ± 0.021 ^a^	0.486 ± 0.054 ^b^	0.118 ± 0.024 ^a^
D12	0.145 ± 0.022 ^a^	0.169 ± 0.010 ^a^	0.544 ± 0.075 ^b^	0.122 ± 0.025 ^a^
D13	0.160 ± 0.015 ^a^	0.179 ± 0.007 ^a^	0.555 ± 0.079 ^b^	0.122 ± 0.020 ^a^
D14	0.159 ± 0.016 ^a^	0.184 ± 0.007 ^a^	0.578 ± 0.072 ^b^	0.133 ± 0.023 ^a^

Note: One-way ANOVA was performed for each day. Different superscript letters within the same row indicate significant differences (*p* < 0.05).

**Table 8:** Fitting curve equations of photosynthetic pigments in four types of microalgae.

Species	Photosynthetic Pigment Contents	Formula	*R*^2^ Value
*K. cornuta* HY-1	Chl-a	Y = 0.11135·e^(0.10201x)^	0.9631
	Chl-b	Y = 0.08162·e^(0.119889x)^	0.9605
	Car	Y = 0.0473·e^(0.09148x)^	0.9810
*D. abundans* HY-1	Chl-a	Y = e^(−1.90336+0.10404x)^	0.9843
	Chl-b	Y = −3.35125 + 3.46307·e^(0.00953x)^	0.9943
	Car	Y = −27.05434 + 27.07746·e^(0.0004x)^	0.9944
*C.* sp. HY-1	Chl-a	Y = 3.76551 − 3.72962·e^(−0.03423x)^	0.9929
	Chl-b	Y = −15.9391 + 16.05441·e^(0.000418x)^	0.9865
	Car	Y = 2.56332 − 2.59121·e^(−0.01975x)^	0.9902
*O.* sp. HY-1	Chl-a	Y = 6.28087 − 6.24639·e^(−0.0063x)^	0.9907
	Chl-b	Y = 0.83113 − 0.84342·e^(−0.066x)^	0.9968
	Car	Y = 0.25316 − 0.2613·e^(−0.07366x)^	0.9685

Note: Chl-a, chlorophyll a; Chl-b, chlorophyll b; Car, carotenoids. Y: Photosynthetic pigment contents, μg·mL^−1^; *x*: culture time, d.

**Table 9:** Effect of different chitosan concentrations on zeta potential.

Microalgae Species	Chitosan							
	Zeta Potential (mV)				pH			
	0 g·L^−1^	0.5 g·L^−1^	1 g·L^−1^	1.5 g·L^−1^	0 g·L^−1^	0.5 g·L^−1^	1 g·L^−1^	1.5 g·L^−1^
*K. cornuta* HY-1	−22.72 ± 0.76 ^b^	−6.28 ± 0.18 ^a^	5.93 ± 0.08 ^a^	5.68 ± 0.16 ^a^	7.68 ± 0.22 ^c^	6.22 ± 0.33 ^b^	5.33 ± 0.27 ^a^	4.94 ± 0.30 ^a^
*D*. *abundans* HY-1	−24.34 ± 1.37 ^b^	−5.48 ± 0.20 ^a^	4.53 ± 0.09 ^a^	4.21 ± 0.12 ^a^	7.8 ± 0.36 ^b^	6.17 ± 0.37 ^a^	5.47 ± 0.32 ^a^	5.22 ± 0.28 ^a^
*C*. sp. HY-1	−23.97 ± 0.56 ^b^	−5.64 ± 0.17 ^a^	5.07 ± 0.06 ^a^	5.23 ± 0.13 ^a^	7.53 ± 0.25 ^c^	6.15 ± 0.35 ^b^	5.31 ± 0.31 ^ab^	5.02 ± 0.32 ^a^
*O*. sp. HY-1	−27.65 ± 0.63 ^b^	−5.16 ± 0.22 ^a^	4.47 ± 0.13 ^a^	4.50 ± 0.15 ^a^	7.5 ± 0.34 ^c^	6.47 ± 0.29 ^b^	5.60 ± 0.23 ^ab^	5.35 ± 0.27 ^a^

Note: One-way ANOVA was performed for each microalgae. Different superscript letters within the same row indicate significant differences (*p* < 0.05).

**Table 10:** Effect of different alkaline flocculant concentrations on zeta potential.

Microalgae Species	Zeta Potential (mV)						pH					
	NaOH (g·L^−1^)			Ca(OH)_2_ (g·L^−1^)			NaOH (g·L^−1^)			Ca(OH)_2_ (g·L^−1^)		
	0.5	1	1.5	0.5	1	1.5	0.5	1	1.5	0.5	1	1.5
*K. cornuta* HY-1	−25.33 ± 0.21 ^b^	−37.88 ± 0.24 ^a^	−37.94 ± 0.25 ^a^	−1.46 ± 0.21 ^c^	−0.52 ± 0.18 ^d^	0.83 ± 0.20 ^cd^	10.71 ± 0.16 ^ab^	11.68 ± 0.19 ^c^	11.74 ± 0.18 ^c^	10.56 ± 0.14 ^a^	11.27 ± 0.21 ^bc^	11.36 ± 0.17 ^c^
*D*. *abundans* HY-1	−23.27 ± 0.08 ^b^	−31.84 ± 0.21 ^a^	−33.31 ± 0.21 ^a^	−1.12 ± 0.21 ^c^	−0.95 ± 0.22 ^c^	1.03 ± 0.21 ^c^	12.00 ± 0.23 ^a^	12.41 ± 0.27 ^a^	12.51 ± 0.25 ^a^	11.87 ± 0.22 ^a^	11.90 ± 0.28 ^a^	11.98 ± 0.20 ^a^
*C*. sp. HY-1	−22.44 ± 0.26 ^b^	−33.77 ± 0.18 ^a^	−33.38 ± 0.18 ^a^	−0.82 ± 0.19 ^c^	−0.59 ± 0.18 ^c^	0.72 ± 0.18 ^c^	11.83 ± 0.18 ^ab^	12.17 ± 0.23 ^ab^	12.23 ± 0.21 ^b^	11.52 ± 0.15 ^a^	11.79 ± 0.24 ^ab^	12.20 ± 0.19 ^ab^
*O*. sp. HY-1	−25.61 ± 0.24 ^b^	−33.53 ± 0.21 ^a^	−33.05 ± 0.18 ^a^	−0.97 ± 0.26 ^c^	1.07 ± 0.13 ^c^	1.08 ± 0.26 ^c^	11.72 ± 0.14 ^ab^	12.10 ± 0.18 ^ab^	12.19 ± 0.16 ^b^	11.59 ± 0.12 ^a^	11.64 ± 0.20 ^a^	11.77 ± 0.14 ^ab^

Note: One-way ANOVA was performed for each microalgae. Different superscript letters within the same row indicate significant differences (*p* < 0.05).

**Table 11:** Effect of different aluminum salts concentrations on zeta potential.

Microalgae Species	Zeta Potential (mV)						pH					
	KAl(SO_4_)_2_ (g·L^−1^)			Al_2_(SO_4_)_3_ (g·L^−1^)			KAl(SO_4_)_2_ (g·L^−1^)			Al_2_(SO_4_)_3_ (g·L^−1^)		
	0.5	1	1.5	0.5	1	1.5	0.5	1	1.5	0.5	1	1.5
*K. cornuta* HY-1	−4.75 ± 0.14 ^a^	−2.91 ± 0.23 ^b^	1.59 ± 0.20 ^c^	−5.33 ± 0.18 ^a^	−4.74 ± 0.23 ^a^	1.56 ± 0.15 ^c^	4.77 ± 0.11 ^b^	4.40 ± 0.`4 ^ab^	4.12 ± 0.09 ^a^	4.51 ± 0.17 ^ab^	4.17 ± 0.13 ^a^	4.04 ± 0.18 ^ab^
*D*. *abundans* HY-1	−4.69 ± 0.22 ^a^	1.95 ± 0.19 ^b^	1.27 ± 0.14 ^c^	−1.43 ± 0.20 ^c^	1.18 ± 0.19 ^c^	1.67 ± 0.18 ^bc^	4.81 ± 0.12 ^c^	4.45 ± 0.16 ^abc^	4.26 ± 0.10 ^ab^	4.64 ± 0.19 ^bc^	4.21 ± 0.13 ^ab^	4.05 ± 0.15 ^a^
*C*. sp. HY-1	0.73 ± 0.15 ^ab^	0.86 ± 0.16 ^ab^	0.94 ± 0.13 ^ab^	−1.14 ± 0.17 ^a^	0.48 ± 0.16 ^b^	0.66 ± 0.14 ^ab^	4.76 ± 0.13 ^b^	4.21 ± 0.25 ^ab^	4.13 ± 0.18 ^ab^	4.73 ± 0.31 ^b^	3.92 ± 0.22 ^a^	3.83 ± 0.27 ^a^
*O*. sp. HY-1	−1.92 ± 0.18 ^c^	−1.86 ± 0.18 ^c^	1.19 ± 0.14 ^c^	−5.61 ± 0.21 ^a^	−5.27 ± 0.24 ^a^	3.14 ± 0.24 ^b^	4.74 ± 0.16 ^a^	4.51 ± 0.30 ^a^	4.29 ± 0.10 ^a^	4.58 ± 0.36 ^a^	4.15 ± 0.22 ^a^	4.02 ± 0.21 ^a^

Note: One-way ANOVA was performed for each microalgae. Different superscript letters within the same row indicate significant differences (*p* < 0.05).

**Table 12:** Effect of different iron salts concentrations on zeta potential.

Microalgae Species	Zeta Potential (mV)						pH					
	FeSO_4_ (g·L^−1^)			FeCl_3_ (g·L^−1^)			FeSO_4_ (g·L^−1^)			FeCl_3_ (g·L^−1^)		
	0.5	1	1.5	0.5	1	1.5	0.5	1	1.5	0.5	1	1.5
*K. cornuta* HY-1	−5.64 ± 0.25 ^a^	−5.47 ± 0.25 ^a^	−5.39 ± 0.17 ^a^	−3.25 ± 0.22 ^c^	1.27 ± 0.17 ^d^	2.09 ± 0.16 ^b^	6.48 ± 0.43 ^a^	6.15 ± 0.38 ^a^	6.09 ± 0.37 ^a^	3.79 ± 0.44 ^b^	3.52 ± 0.40 ^b^	3.45 ± 0.43 ^b^
*D*. *abundans* HY-1	−2.75 ± 0.25 ^bc^	−2.34 ± 0.25 ^bc^	−2.16 ± 0.21 ^c^	−1.48 ± 0.17 ^d^	2.44 ± 0.20 ^b^	4.82 ± 0.22 ^a^	6.64 ± 0.12 ^c^	6.35 ± 0.14 ^ab^	6.12 ± 0.10 ^b^	3.76 ± 0.14 ^a^	3.65 ± 0.16 ^a^	3.58 ± 0.12 ^a^
*C*. sp. HY-1	−2.36 ± 0.21 ^b^	−1.38 ± 0.20 ^c^	−1.17 ± 0.24 ^cd^	−0.61 ± 0.15 ^d^	1.33 ± 0.14 ^c^	3.75 ± 0.19 ^a^	6.60 ± 0.12 ^c^	6.36 ± 0.14 ^c^	6,17 ± 0.09 ^c^	3.82 ± 0.16 ^b^	3.69 ± 0.18 ^ab^	3.24 ± 0.15 ^a^
*O*. sp. HY-1	−2.47 ± 0.20 ^b^	−2.33 ± 0.30 ^b^	1.41 ± 0.18 ^c^	−1.02 ± 0.20 ^c^	0.76 ± 0.16 ^c^	4.58 ± 0.18 ^a^	6.87 ± 0.11 ^b^	6.53 ± 0.14 ^b^	6.39 ± 0.13 ^b^	3.90 ± 0.18 ^a^	3.62 ± 0.15 ^a^	3.53 ± 0.18 ^a^

Note: One-way ANOVA was performed for each microalgae. Different superscript letters within the same row indicate significant differences (*p* < 0.05).

## Data Availability

The data that support the findings of this study are available from the corresponding author upon reasonable request.

## Abbreviations

*K. cornuta* HY-1	*Kirchneriella cornuta* HY-1
*D. abundans* HY-1	*Desmodesmus abundans* HY-1
*C.* sp. HY-1	*Coccomyxa* sp. HY-1
*O.* sp. HY-1	*Oocystis* sp. HY-1
NH_4_^+^-N	Ammonia nitrogen
NO_2_-N	Nitrite nitrogen
TP	Total phosphorus
RAS	Recirculating aquaculture system
Chl-a	Chlorophyll a
Chl-b	Chlorophyll b
Car	Carotenoids
ALE	Adaptive laboratory evolution
